# Recent Trends in the Application of Cellulose-Based Hemostatic and Wound Healing Dressings

**DOI:** 10.3390/jfb16050151

**Published:** 2025-04-23

**Authors:** Clemence Futila Bukatuka, Bricard Mbituyimana, Lin Xiao, Abeer Ahmed Qaed Ahmed, Fuyu Qi, Manjilla Adhikari, Zhijun Shi, Guang Yang

**Affiliations:** 1 Department of Biomedical Engineering, College of Life Science and Technology, Huazhong University of Science and Technology, Wuhan 430074, China; clemsbuka326@gmail.com (C.F.B.); bricard7@yahoo.com (B.M.); qi_fy@foxmail.com (F.Q.); true1.truth2@gmail.com (M.A.); shizhijun@hust.edu.cn (Z.S.); 2 School of Biomedical Engineering, Sun Yat-sen University, Shenzhen 518107, China; 3 Biochemistry Unit, Department of Molecular Medicine, University of Pavia, 27100 Pavia, Italy; ahmed01abeer01@yahoo.com; 4 Organ Transplantation Clinical Medical Research Center of Hubei Province, Wuhan 430030, China

**Keywords:** cellulose dressings materials, hemostasis control and management, wound healing enhancement, bioactivity improvement, biocompatibility and cytocompatibility

## Abstract

Rapid hemostasis and wound healing are crucial severe trauma treatment. Natural mechanisms often prove insufficient, spurring research for innovative biomaterials. This review focuses on cellulose-based materials, which are promising due to their absorbency, biocompatibility, and processability. The novelty lies in exploring how these materials promote clotting and tissue regeneration. They operate via extrinsic and intrinsic mechanisms. Extrinsically, they create a matrix at the wound to activate coagulation; intrinsically, they maintain clotting factors. Additionally, they aid healing through physical, chemical, and biological means, such as maintaining moisture, incorporating antimicrobial agents, and stimulating cell activity. The innovative fabrication strategies include material selection and chemical modification. Techniques like oxidation enhance performance. Structural engineering methods like freeze-drying and 3D printing optimize porosity and alignment. Cellulose-based dressings are versatile and effective in various forms. They address different wound needs and show benefits like rapid coagulation and tissue repair. This review also covers challenges and future trends, emphasizing the need to enhance mechanical properties and biodegradability. Further, new technologies offer potential improvements to the nanocomposites. Overall, continued research on cellulose-based dressing is vital, and unlocking their potential could revolutionize wound care, providing suitable solutions for trauma management.

## 1. Introduction

In the global healthcare landscape, many premature deaths result from emergencies where stopping bleeding and speeding up wound healing can save lives [1]. Although the body’s inbuilt hemostatic and self-healing mechanisms valiantly attempt to kick into gear, severe trauma frequently slips through these natural safeguards, resulting in staggeringly high mortality rates that demand urgent solutions [2]. This urgent medical need has driven the search for advanced materials with dual-pronged potency in hemostasis and wound healing, which have emerged as a major focus of radical innovation, revolutionizing modern medicine [3].

Hemostatic materials have embarked on an extraordinary evolutionary journey. Unlike traditional materials that just soak up blood, today’s advanced materials actively speed up clotting. They use potent bioactive compounds and nano-engineered particles to create a strong barrier against bleeding, often in seconds [4]. Wound healing materials have also undergone a metamorphosis. They now deploy a dazzling array of smart antibacterial arsenals, harnessing everything from precision-engineered nanoparticles that target specific pathogens to gene-edited bacteriophages that can selectively wipe out harmful bacteria without disrupting the beneficial microbiome. Immunomodulation has reached new heights of sophistication, with biomaterials that can fine-tune the body’s immune response, ensuring the perfect balance between inflammation to jumpstart healing and prevent overactive immune reactions that could impede recovery. Moisture retention is no longer a simple matter of just keeping the wound moist; it is about engineering hydrogels with self-regulating water-release mechanisms that mimic the body’s natural hydration cycles, creating an optimal microenvironment for cell proliferation and tissue regeneration [5]. This includes utilizing materials that can induce stem cells to act using bioengineered signals and growth factor gradients, reducing wound healing times by up to 50% or more in some cases. Some studies have reported up to 80% reduction in wound healing time in preclinical models [6,7]. The interplay between hemostasis and wound healing shows there is a growing need for biomaterials that can address both mechanisms simultaneously. The multifunctional materials aim to bridge the gap between these two processes, offering integrated and innovative therapeutic solutions [8,9].

Cellulose, a simple yet powerful biodegradable polysaccharide found abundantly in plants and bacteria, has catapulted to the forefront of materials science. It absorbs large amounts of blood, is biocompatible with human tissue, and shows ease of processing, unlocking endless design possibilities [10]. Plant-derived cellulose offers purity and stability, ensuring that medical-grade dressings can be produced with consistent quality that was previously unattainable. Bacterial cellulose, meanwhile, stands out for its unique properties, such as exceptional water and moisture retention and a 3D porous network that acts like a scaffold, creating an ideal environment for cell growth [11,12]. Nanostructured cellulose and modified derivatives, such as carboxymethylcellulose, have unleashed innovation. They do not just enhance the hemostatic and wound healing properties; they shatter existing paradigms. By functionalizing with bioactive molecules, we are now seeing dressings that can release drugs on demand, with antibacterial properties that can wipe out superbugs. This functionalization strategy is unlocking entirely new ways to treat wounds that were once thought impossible [13,14].

Cellulose-based wound dressings, riding this wave of innovation, have devised a groundbreaking mechanism. In fluid balance management, they have pioneered the use of smart membranes that can sense wound hydration needs and adjust their permeability in real time. These advanced systems often incorporate stimuli-responsive polymers or hydrogel components that swell or contract depending on moisture levels, thus maintaining an optimal healing environment. This adaptive functionality helps prevent excessive dryness and maceration, contributing to faster and more efficient healing [15]. Their infection prevention strategies are revolutionary, deploying self-cleaning surfaces coated with antimicrobial peptides that can repel bacteria and microencapsulated antibiotics that can release a burst of medicine when bacteria are detected, keeping pathogens at bay more effectively than ever before. In tissue regeneration, they are harnessing the power of bioengineering to create scaffolds that mimic the body’s extracellular matrix with astonishing precision, using 3D printing to build custom structures that can guide cell growth and differentiation, addressing a wide range of wound types with a level of versatility and adaptability that is game-changing [16,17,18]. These dressings, with their high absorbency, shapeable nature, biocompatibility, and all-encompassing versatility, have become the solution of choice for a diverse range of wounds, as vividly demonstrated in Table 1 [19,20].

Inspired by the wealth of evidence spotlighting the extraordinary potential properties of cellulose-based dressings in conquering the challenges of hemostasis and wound healing, this review embarks on a journey to explore the latest and most innovative advances in this domain. It explores the complex processes behind hemostasis and wound healing from cutting-edge clotting pathways to innovative chemical, physical, and biological healing mechanisms that are transforming treatment approaches. It then delves into the revolutionary fabrication and design procedures that are birthing these materials, such as pioneering chemical modification and functionalization techniques that push the boundaries of what materials can do; layer-by-layer assembly that creates structures of unimaginable complexity and functionality; freeze-drying that preserves the delicate architectures of these materials while enhancing their performance; electrospinning that weaves nanofibers into fabrics with remarkable properties; and 3D printing that customizes wound dressings to fit the unique contours of each wound. This review further expands its scope to unpack their broad biomedical and clinical applications, exploring how these innovative wound dressings are transforming patient care. Finally, it fearlessly confronts existing challenges and maps out a bold future direction, with the overarching goal of maximizing clinical impact and ushering in a new era of wound management defined by innovation and excellence.

### 1.1. Stages of the Hemostasis Process

**Figure 1:**
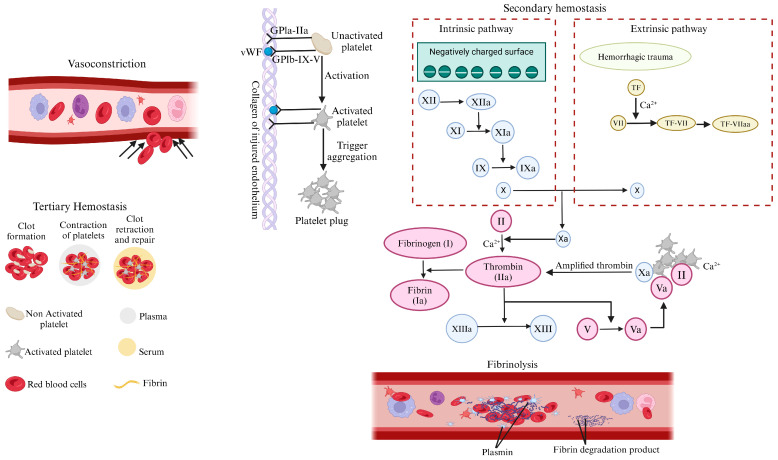
The mechanism of hemostasis and elements involved in coagulation.

Hemostasis is a multi-step process that stops bleeding and facilitates wound repair by forming a hemostatic plug to seal the injured vessel [33] (Figure 1). The initial step begins with vasoconstriction, which occurs immediately after vascular injury; blood vessels constrict to reduce blood flow and minimize blood loss [34]. This is followed by the next step, known as primary hemostasis, which involves platelets adhering to exposed collagen at the injury site becoming activated and aggregating, forming a temporary platelet plug. This plug provides an initial seal and initiates the clotting process [35]. The receptors’ interactions, such as GPIa-IIa and GPIb-IX-V, facilitate platelet activation and aggregation [36]. Subsequently, the coagulation cascade is also known as secondary hemostasis [37]. This stage involves a complex sequence of clotting factors that convert fibrinogen into fibrin threads, which weave through the platelet plug, stabilizing it into a firm clot [38]. This process includes both extrinsic and intrinsic pathways and reinforces vascular integrity [39]. This is then followed by clot retraction, also known as tertiary hemostasis [40]. This process promotes platelet contraction, fibroblast incorporation, and pulling wound edges together, or wound edge approximation, aiding in tissue repair and forming a stable, cohesive clot [41]. Finally, fibrinolysis occurs. Once the vessel has healed sufficiently, plasmin degrades the fibrin into soluble fragments, effectively dissolving the clot to restore normal blood flow and prevent unwanted clotting [42].

### 1.2. Phases of Wound Healing

**Figure 2:**
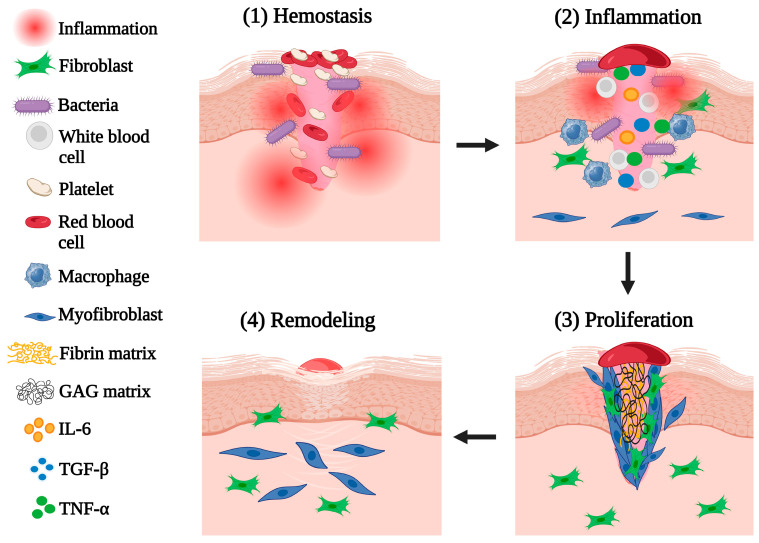
The stages of wound repair and the key cellular components involved.

Similarly, wound healing involves four distinct phases that work collectively to repair tissue damage and restore skin integrity [9] (Figure 2). Hemostasis is the initial stage that stops bleeding by forming a clot at the wound site, which also serves as a matrix for subsequent phases of healing [43]. The inflammation phase follows, which is the second stage and is marked by the influx of immune cells, primarily neutrophils and macrophages, that arrive to eliminate bacteria, clear debris, and prevent infection [44]. This stage is marked by the release of cytokines, including IL-6 and TNF-alpha, which attract inflammatory cells to the site [45]. Then follows the tertiary stage, the proliferation phase, which involves the formation of new tissue by fibroblasts, endothelial cells, and keratinocytes [45]. Fibroblasts produce collagen, endothelial cells promote angiogenesis, and keratinocytes facilitate re-epithelialization. TGF-beta plays a critical role in activating fibroblasts and promoting tissue reconstruction [46]. The last stage, remodeling, follows: During this phase, type III collagen is replaced by type I collagen to increase tissue strength, with fibroblasts secreting enzymes to organize the collagen matrix [47]. Myofibroblasts contribute to wound contraction, while unnecessary cells are eliminated by apoptosis. This phase leads to mature and strengthened scar tissue [48].

## 2. Cellulose-Based Composites as the Best Candidate for Hemostasis and Wound Healing

**Figure 3:**
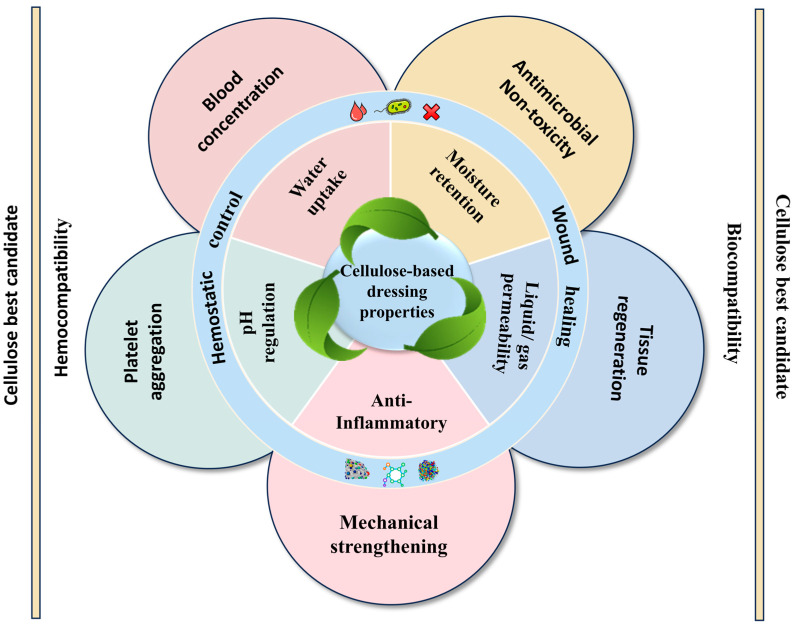
Functional properties of cellulose-based composites for hemostasis and wound healing.

As an exemplary hemostatic material, cellulose-based composites present a host of remarkable attributes that render them highly suitable for a wide range of clinical requirements [49]. Their ease of manipulation, rapid clot-forming potential, and excellent biocompatibility set them apart, carving out a promising niche in hemostasis and wound healing applications. This is underpinned by their customizable chemical, mechanical, and biological characteristics, as depicted in Figure 3 [50,51].

Cellulose-based composite dressings operate through multiple mechanisms to achieve swift hemostasis. They can concentrate blood, effectively gathering red blood cells and other components in a localized area. This is in contrast to some traditional dressings that may allow blood to disperse more widely, preventing clot formation. By inducing hemocyte aggregation, these dressings mimic the body’s natural clotting initiation process. The fibers and additives within the composites act as nucleation sites, prompting platelets and other cells to cluster together. This is far more efficient than dressings that lack such an aggregation function, as it accelerates the coagulation cascade [52]. The fluid absorption capacity of these composites is also crucial. Compared to materials with lower absorbency, they can quickly absorb blood and wound exudates, creating a more conducive environment for platelet activation. The mechanical strength they possess further aids in this process, providing a stable structure that encourages platelet adhesion and activation of coagulation factors [53].

Chitosan, a natural polymer, forms a synergistic duo when combined with cellulose. Chitosan’s ability to form a gel matrix upon contact with blood is a game changer. It acts as a molecular magnet, attracting platelets and accelerating clot formation. In comparison to cellulose-based dressings without chitosan, the clotting time can be significantly reduced. Commercial dressings such as Chito-Sorb have capitalized on this cellulose-chitosan synergy with great effect in stemming bleeding in acute wounds [54,55]. Another polysaccharide, alginate sourced from seaweed, impacts its unique ion-exchange capabilities to cellulose dressings. Upon contact with wound exudate, it undergoes transformation, forming a gel that creates a moist, protective barrier. This not only keeps the wound hydrated but also accelerates the clotting process. Research by Ouyang et al. demonstrated that wounds treated with alginate-cellulose dressings healed faster in skin defect models than those treated with conventional dressings [56].

Minerals such as kaolin and zeolites have also been incorporated into cellulose with great success. Kaolin, for one, activates coagulation factors XII and XI upon interacting with blood. This direct activation of the coagulation cascade is a distinct advantage over dressings that do not contain kaolin. Studies have shown that kaolin-impregnated cellulose dressings outperform standard cotton gauze in terms of hemostatic efficacy, particularly in managing severe hemorrhages. In fact, their FDA approval for trauma and surgical applications further underscores their clinical superiority in critical bleeding [57]. Zeolites, with their microporous structure, offer a dual benefit. They not only absorb fluids, much like other absorbent materials, but also activate the intrinsic coagulation pathway, a function that few other additives possess. Additionally, both kaolin and zeolites exhibit antimicrobial activity, providing an added layer of defense against a wide spectrum of microorganisms [58].

Synthetic polymers such as polyvinyl alcohol (PVA) also find their place in cellulose-based composites. When integrated, they enhance the mechanical durability and moisture retention of dressings without compromising their hemostatic capabilities. In contrast to composites without PVA, those containing it can better withstand the rigors of wound care, preserving their integrity over time. Research has indicated that PVA-cellulose hydrogels promote wound closure and tissue regeneration, particularly in challenging scenarios such as diabetic wounds, where structural resilience is vital [59].

Collagen-enriched cellulose dressings serve as a scaffold for cell migration and tissue regeneration. In chronic and diabetic wounds, where the body’s natural repair mechanisms are often slow, these dressings can make a significant difference. Compared to collagen-free dressings, they accelerate wound healing and angiogenesis, providing the necessary structural and cellular support [60].

To combat infection, cellulose dressings are frequently enriched and augmented with silver nanoparticles or hypochlorous acid. Silver nanoparticles disrupt bacterial cell membranes, triggering a broad-spectrum antibacterial effect that can even overpower antibiotic-resistant strains. Studies have spotlighted that silver nanoparticle-cellulose dressings are highly effective in inhibiting common pathogens such as *Staphylococcus aureus* and *Pseudomonas aeruginosa*. Hypochlorous acid, on the other hand, takes a broader range of microorganisms, reducing inflammation and the risk of infection [61].

Oxidized cellulose, produced by treating cellulose with agents like nitric acid, introduces functional groups that enhance its hemostatic properties. It forms stable clots at bleeding sites in the surgical setting, reducing postoperative bleeding more effectively than non-oxidized cellulose. When combined with synthetic or natural materials, oxidized cellulose composites display enhanced adhesive properties and improved hemostatic performance, making them invaluable in complex surgical wounds and mucosal injuries [62].

In conclusion, incorporation of natural and synthetic additives into cellulose dressings creates a multifunctional platform that addresses diverse hemostasis and wound healing needs. Their biocompatibility, antimicrobial prowess, and ability to foster cell regeneration make them optimal choices for wound management across a spectrum of clinical scenarios, from acute trauma to chronic wound care. Future research could focus on further optimization of these composites, perhaps by comparing different additive combinations to identify the most potent formulations or by exploring how to fine-tune the release kinetics of antimicrobial agents to maximize their efficacy.

## 3. Mechanisms of Cellulose-Based Dressings for Hemostasis and Wound Healing

### 3.1. Hemostasis Mechanisms of Cellulose-Based Dressing Materials

Cellulose dressings have emerged as highly effective hemostatic agents, leveraging both external and intrinsic mechanisms to halt bleeding and stabilize wounds [63].

#### 3.1.1. External Mechanisms

**Figure 4:**
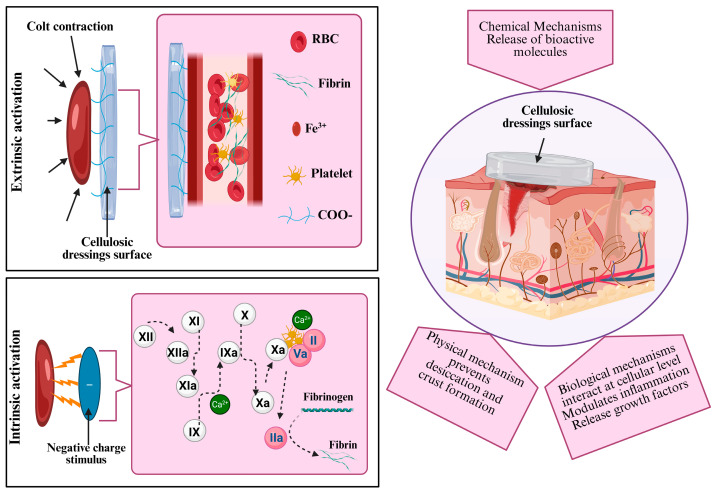
Visual diagram showing the mechanisms of cellulose dressings in coagulation and wound repair.

The external mechanisms of cellulose-based hemostasis revolve primarily around extrinsic factors, which are characteristics of dressings that interact with the external environment to promote hemostasis [64]. When in contact with blood, cellulose dressings instantaneously form a dense matrix at the bleeding site. This is in contrast to some other types of dressings that may not be able to create such a structured and effective barrier. The dense matrix plays a crucial role in activating the extrinsic coagulation pathway, jumpstarting the body’s natural clotting process [65]. The large surface area of cellulose fibers is another significant advantage. Compared to dressings with smaller fiber surface areas, cellulose dressings can absorb a substantial amount of blood and exudates. This absorption capacity is not only beneficial for maintaining a clean wound environment but also facilitates platelet activation and aggregation. In fact, the large and extensive surface area provides numerous sites for platelets to adhere and interact, similar to a scaffold that promotes their congregation and assembly [66]. This, in turn, accelerates the coagulation cascade, leading to the rapid formation of a stable clot that effectively seals the wound. For instance, cellulose fibers act as a catalyst for platelet interaction, hastening the creation of a hemostatic plug much more effectively than some traditional dressings that lack such fiber-induced platelet activation capabilities [19]. Illustrations of the mechanisms of cellulose-based dressings for hemostasis and wound healing are presented in Figure 4 and Table 2. Each section visually depicts the external, intrinsic, physical, chemical, and biological mechanisms at work in wound management, detailing the role of cellulose on the coagulation pathway and cell migration in enhancing healing.

#### 3.1.2. Intrinsic Mechanisms

Simultaneously, the intrinsic mechanisms of cellulose dressings offer additional layers of hemostatic support. These dressings form a physical barrier that serves multiple functions. Unlike wound dressings, which do not have the ability to retain clotting factors, cellulose dressings can effectively maintain clotting factors at the wound site. This is achieved through the formation of a cohesive adhesive layer that acts like a molecular net, capturing and holding platelets and other clotting factors in place. This local concentration of clotting components bolsters the clot formation process, ensuring the clot remains stable and effective [67,68]. The porous structure of cellulose is another key feature. Compared to non-porous dressings, the porosity allows for efficient oxygen exchange. This promotes a healthier wound environment, as oxygen is essential for cellular metabolism and tissue repair. Adequate oxygenation can prevent the development of anaerobic conditions that might otherwise lead to infection or delayed wound healing [69].

Moreover, cellulose materials can initiate coagulation via contact activation pathways. This is a unique property that distinguishes them from many other dressing materials. By stimulating enzymes such as clotting factors, they enhance the coagulation process in a manner that is both direct and efficient. Some other dressings may rely solely on passive absorption or simple physical barriers, while cellulose dressings actively engage in the biochemical aspects of coagulation, providing a more comprehensive hemostatic solution [70]. The combination of these external and intrinsic mechanisms makes cellulose dressings a formidable option in the realm of hemostasis. Their ability to work synergistically at multiple levels, from physical barrier formation to biochemical activation, positions them as a leading choice for stopping bleeding and promoting wound stability. Future research could focus on optimizing these mechanisms, perhaps by comparing different cellulose-based formulations to identify the most effective clotting enhancers or exploring how to fine-tune the porosity to maximize oxygen exchange and tissue repair.

### 3.2. Wound Repair Mechanisms of Cellulose-Based Dressing Materials

Beyond hemostasis, cellulose dressings play a crucial and active role in promoting wound healing through a combination of physical, chemical, and biological mechanisms, as detailed in Table 2 and Figure 4.

#### 3.2.1. Physical Mechanisms

The physical mechanisms by which cellulose dressings contribute to wound healing are of paramount importance. Their absorbency is a key aspect. In comparison to dressings that are poorly absorbent or do not manage moisture effectively, cellulose dressings can prevent excessive moisture accumulation as well as desiccation. Excessive moisture can create a breeding ground for bacteria and disrupt the normal tissue repair process, while desiccation can lead to drying of the wound bed and impede the growth of new tissue [71]. By maintaining an optimal moisture balance, these dressings facilitate autolytic debridement. Unlike traditional dressings, which may require mechanical debridement methods, cellulose dressings can gently break down necrotic tissue by keeping the wound environment moist. This softening of necrotic tissue makes it easier and more natural to remove, which in turn accelerates the formation of new tissue. Moreover, the moist environment created by cellulose dressings provides a favorable condition for re-epithelialization. In contract to dry dressings that may cause the wound to crust over, which can delay the healing process and lead to uneven scarring, cellulose dressings promote a smoother healing process by allowing epithelial cells to migrate and cover the wound bed more effectively [72].

#### 3.2.2. Chemical Mechanisms

When it comes to chemical mechanisms, some cellulose-based dressings (CBDs) incorporate bioactive agents, presenting a distinct advantage over dressings without such additives. For instance, some incorporate silver nanoparticles, while others use essential oils [61,73]. These bioactive agents have potent antimicrobial properties. Compared to dressings that lack antimicrobial components and are thus more prone to bacterial colonization, these cellulose-based dressings can effectively inhibit the growth of pathogenic bacteria. By reducing the bacterial load in the wound, they lower the risk of infection and create a more sterile wound environment [74].

The chemical modification of cellulose-based dressings not only prevents infection but also optimizes the overall wound healing environment. The sustained release of growth factors and antimicrobial activity from these dressings is another important aspect. Compared with dressings that release these substances in an uncontrolled or a short-lived manner, cellulose dressings can provide a more consistent and prolonged supply of these beneficial agents. This promotes healing and reduces the risk of wound-related complications, such as delayed healing or re-infection [6].

#### 3.2.3. Biological Mechanisms

At the cellular level, biological mechanisms come into play. Cellulose-based dressings have a notable impact on the behavior of critical wound-healing cells, including keratinocytes, fibroblasts, and endothelial cells [75]. In contrast to dressings that do not interact favorably with these cells or may even disrupt their normal functions, cellulose-based dressings encourage the migration and proliferation of these cells. For example, they may provide an appropriate and suitable substrate or signaling cues that prompt keratinocytes to migrate across the wound bed for re-epithelialization and fibroblasts to produce collagen for tissue strengthening.

Furthermore, they play a regulatory role in inflammatory responses. By modulating the levels of cytokines, they decrease the production of pro-inflammatory cytokines while increasing the release of anti-inflammatory cytokines. This differs from dressings that may not have such regulatory capabilities and could potentially allow excessive or prolonged inflammation to occur. The ability of cellulose dressings to create an environment conducive to tissue repair by reducing prolonged inflammation leads to a more balanced and efficient wound healing process [76]. In addition, cellulose-based dressings can promote angiogenesis, which is vital for supplying nutrients and oxygen to the wound area. In comparison to dressings that do not promote or even hinder new blood vessel formation, these dressings facilitate the growth of new blood vessels, improving the delivery of essential nutrients and promoting better tissue regeneration. Overall, the multifaceted mechanisms by which cellulose dressings work make them a valuable asset in wound healing, with each mechanism working in concert to optimize the wound healing process and improve patient outcomes [77].

**Table 2:** Mechanisms of cellulose-based dressings in hemostasis and wound healing.

Mechanism	Description	Ref.
Exterior mechanisms	Large surface area of fibers forms a dense matrix Absorbs fluid and exudate	[65,66]
Interior mechanisms	Physical barrier Potential activation of FXII Tigered intrinsic pathway	[67,70]
Physical mechanisms	Fluid absorbance, desiccation, crust formation, and necrotic tissue removal	[78,79]
Mechanical mechanisms	Release bioactive (growth factors, cytokines) Provide bactericide effect	[61,80,81]
Biological mechanisms	Interact at cellular level Modulate inflammation, migration, and proliferation of key cells (keratinocytes, fibroblasts, endothelial cells).	[77,82,83]

## 4. Fabrication Design Strategies for Novel Cellulose-Based Hemostatic and Wound Dressing Materials

Material design is crucial, as it determines the structures, shapes, properties, and performances of materials. Innovative solutions in wound care have led to significant advancements in the development of cellulose-based composite dressings, offering tailored approaches to hemostasis and wound healing. This burgeoning field integrates diverse preparation methods and designs to create dressings that not only staunch bleeding but also foster optimal conditions for tissue regeneration.

### 4.1. Fabrication Design Strategies for Hemostatic Cellulose Dressings

Designing an effective hemostatic cellulose-based dressing requires a thoughtful combination of materials, microstructures, and bioactive components to optimize hemostasis, antimicrobial protection, and tissue regeneration. This approach exploits the design parameters mentioned in this section, which include material selection criteria, chemical modification, optimization of manufacturing structure, functional parameters, and additional manufacturing design based on recent innovative studies [1].

#### 4.1.1. Material Selection and Chemical Modifications

Material selection constitutes a fundamental step in the development of biomedical products, particularly for hemostatic and wound healing applications. The ideal material must meet a specific set of biological, physical, and mechanical requisites that are fine-tuned to its intended function. Biocompatibility stands as a foremost and non-negotiable criterion. The material is required to refrain from triggering any immune responses or exhibiting toxicity while concurrently promoting cell adhesion, proliferation, and, ultimately, tissue regeneration. Moreover, it must interact harmoniously and effectively with blood components to improve hemostasis without precipitating adverse consequences such as thrombosis or excessive coagulation, which could lead to life-threatening complications [84].

Mechanical properties assume a significant role in dictating the performance of the material. This is a distinct advantage over materials that lack such adaptability and may not provide adequate coverage or protection. It must also maintain its structural integrity under the physiological conditions prevalent during the healing process. High porosity is another critical attribute, as it facilitates cell infiltration and gas exchange, which are essential for proper generation of tissue. The swelling capacity of the material is equally indispensable, as it aids in fluid absorption and helps maintain a moist wound environment, which is beneficial for healing, unlike materials that may dry out the wound or cause excessive moisture retention.

Surface properties, such as hydrophilicity or charge, exert a profound influence on platelet adhesion and clotting factor activation, which are pivotal for efficient hemostasis [85]. For example, a hydrophilic surface may attract more water molecules, which in turn can affect the behavior of platelets and clotting factors, either enhancing or inhibiting the hemostatic process depending on the degree of hydrophilicity. In contrast, a hydrophobic surface can repel water and interact differently with blood components. Functional performance mandates that the material swiftly controls bleeding by promoting platelet adhesion and facilitating interactions with clotting factors. This is a key differentiator from materials that may not have such active mechanisms for promoting hemostasis.

Control of degradation is of utmost importance for biodegradable materials. The material must degrade in a controlled manner to ensure that it promotes healing without releasing harmful byproducts. This is in contrast to some biodegradable materials that may degrade too quickly or release toxic substances during the degradation process. Additionally, the material must be cost-effective and adaptable to scalable manufacturing processes, such as freeze-drying or extrusion. This is essential for large-scale production and commercialization [86].

**Figure 5:**
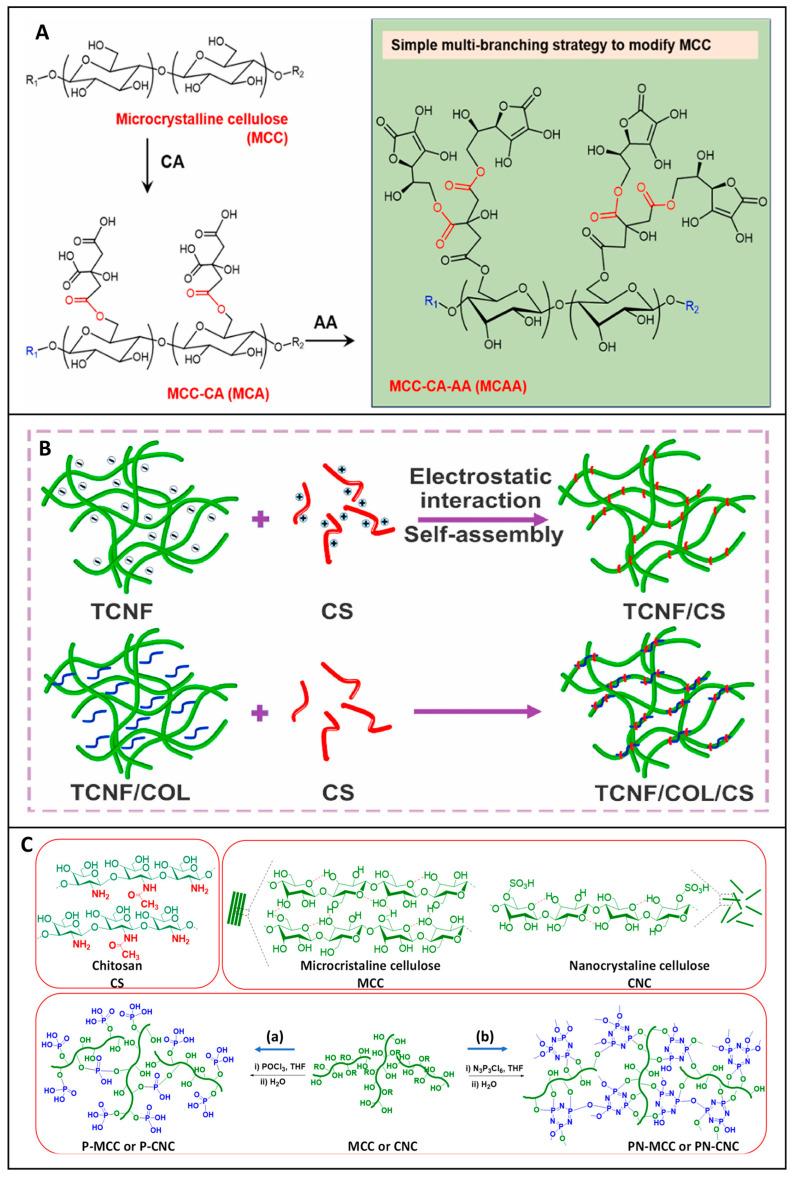
Engineered cellulose-based hemostatic materials through targeted chemical modifications. (**A**) Surface modification of carboxylation of microcrystalline cellulose using citric and ascorbic acid to produce multibranched with enhanced hemostatic performance. Reprinted from Ref. [90]. (**B**) Structure composition and mechanism of a TEMPO oxidized nanofibril composite hemostasis by electrostatic self-assembly method. Reprinted from Ref. [89]. (**C**) Synthesis of phosphorylated cellulose-chitosan films with improved transparency, hemostasis, and biocompatibility. (a, b) Two phosphorylating agents (POCl_3_ or N_3_P_3_Cl_6_) enables the synthesis of two types of phosphorylated cellulose: P-MCC/P-CNC and PN-MCC/PN-CNC. Reprinted from Ref. [91].

To meet these multiple demands, researchers have turned to chemical modifications to enhance the performance of materials. Chemical modifications are integral in augmenting the inherent properties of base materials like cellulose, optimizing their biological and functional performance. Oxidation is a commonly employed modification that introduces aldehyde groups, enhancing material absorbency, platelet interaction, and clotting efficiency while also providing antimicrobial functionality [87]. Oxidized cellulose, for instance, not only expedites rapid coagulation but also provides antimicrobial activity, making it a preferred choice for surgical hemostatic applications [88]. Liu et al. demonstrated that TEMPO Oxidized nanocellulose (TOCN)-based aerogels, combined with collagen (COL) and chitosan (CS), exhibited rapid blood clotting and strong tissue adhesion in vivo, making them excellent candidates for severe bleeding control. The TCNF/COL/CS aerogel dressing was designed using a dual approach: (1) electrostatic self-assembly between polyanionic TCNF and polycationic CS, and (2) physical encapsulation of collagen in a sandwich-like structure, as shown in Figure 5B. This hierarchical assembly resulted in an aerogel with increased porosity, high water uptake capacity, and strong mechanical cohesion, which are critical factors for hemorrhage management [89].

Carboxylation is another essential chemical modification. The addition of carboxyl groups increases the material’s hydrophilicity and interaction with blood components, leading to an enhanced swelling capacity and overall hemostatic efficacy [90]. Complementing this approach, Li et al. developed a multibranch carboxylated microcrystalline cellulose (MCAA) through sequential modification with citric acid and ascorbic acid, as shown in Figure 5A. This modification aims to improve the hemostatic properties of the cellulose by increasing the carboxyl group content to 9.52%, nearly double that of citric-acid-grafted cellulose (MCA, 4.6%). This chemical modification imparted a negatively charged surface to the cellulose, playing a key role in activating the intrinsic coagulation pathway and facilitating platelet adhesion, essential steps for rapid fibrin clot formation [90].

A particularly innovative modification strategy is the phosphorylation of cellulose, explored by Kedzierska et al. in the development of chitosan nanocomposite films loaded with phosphorylated cellulose. In their study, microcrystalline and nanoscale cellulose, both in native and phosphorylated forms, were incorporated into transparent chitosan films. The goal was to investigate the influence of cellulose size and chemical functionalization on wound healing efficiency and hemostasis. These nanocomposite films effectively shortened clotting times by simultaneously activating both intrinsic and extrinsic coagulation pathways, attributed to phosphate groups enhancing electrostatic interactions with coagulation proteins and cellular components such as platelets. Moreover, these films demonstrated antioxidant activity, minimal cytotoxicity, and no genotoxicity across two relevant cell lines, human dermal fibroblasts and keratinocytes, while promoting their migration, an essential factor in re-epithelialization and wound closure [91].

Together, these studies illustrate how strategic modification of the cellulose surface, whether through oxidation or carboxylation, can fine-tune its electrostatic, absorbent, and interactive properties to meet specific biomedical applications. Phosphorylation adds a new dimension to cellulose functionalization, expanding its potential in hemostatic and wound healing formulations. Its capacity to promote blood coagulation while preserving antioxidant and cell migratory properties is demonstrated. These findings align well with broader strategies, such as the development of Janus-structured materials, like that of Cheng et al., where one surface is designed for rapid hemostasis and the other for tissue regeneration.

#### 4.1.2. Structural Engineering and Microstructure Optimization

The optimization of the microstructure with hemostatic materials holds paramount significance in enhancing both blood absorption and coagulation performance. Among the crucial characteristics, high porosity and precisely controlled particle sizes are key attributes in augmenting fluid uptake and facilitating more effective interaction with coagulation factors. For instance, freeze-drying methodologies have been widely utilized to fabricate cellulose-based aerogels and sponges. These possess soft, porous, and flexible structures that are highly conducive to efficient absorption. Tripathi et al. demonstrated that, by incorporating cellulose nanofibers into sodium alginate chitosan aerogels via lyophilization, the surface area was substantially increased. This led to enhanced blood interaction and accelerated coagulation. In contrast to traditional hemostatic materials with less developed microstructures, these advanced cellulose-based composites offer improved performance [21].

When it comes to particle size optimization, studies on oxidized regenerated cellulose (ORC) powders have provided valuable insights. Zeynep et al. emphasized that smaller particle sizes in ORC materials significantly improved coagulation efficiency by effectively concentrating clotting factors. The innovative design of “lotus pod”-like microstructures further exemplifies the importance of microstructure in hemostasis. This design was able to effectively direct blood into the micropores, thereby improving fibrin generation and promoting rapid clot formation. Unlike traditional hemostatic materials with less optimized microstructures, these ORC materials with tailored particle sizes and unique geometries showcase the potential for improved hemostatic outcomes [92].

Microchannel designs present another novel and promising approach to developing hemostatic materials. Chen et al. developed a sponge (CQTC) composed of quaternized chitosan (QCS) and carboxylated cellulose nanofibers (CCNF) using tannic acid and Cu^2^⁺ as crosslinkers. The sponge exhibited effective hemostasis. Additionally, CQTC demonstrated excellent biodegradability and promoted wound healing, making it a more favorable option compared to some non-biodegradable or less effective hemostatic materials. Its unique microstructure and associated properties make it a strong candidate for clinical applications [93].

Recent advances have also explored the potential of superabsorbent materials. Mahmoodzadeh et al. developed a new superabsorbent material, synthesized via chemical and physical crosslinking methods. This material exhibited a remarkable capacity to absorb blood (60 g/g) and adhere strongly to tissue (~90 kPa). The presence of silica nanoparticles and calcium ions in its structure activated coagulation pathways, leading to a significant reduction in clotting time and blood loss [53].

Functional cellulose sponges and composite dressings with unique microstructural features have been designed to address various types of wounds. Fan et al. utilized gas foaming techniques to fabricate cellulose sponge dressings loaded with chitosan. These sponges demonstrated rapid blood swelling ability, excellent mechanical properties, and potent antimicrobial activity against pathogens such as *E. coli* and *S. aureus*. Their scalability and environmental compatibility further distinguish them from other dressings. In an industrial context, these features make them more attractive for large-scale production compared to materials that may be difficult to scale up or have negative environmental impact [94].

Innovative bio-inspired design fabrications, such as mussel-inspired oxidized sodium alginate/cellulose composite dressings developed by Yu et al., have expanded the horizons of hemostatic materials. With the introduction of aldehyde-modified sodium alginate, tissue adhesion and antibacterial properties were improved, resulting in faster clot formation and reduced blood loss. These bio-inspired approaches combine the advantages of natural principles with synthetic engineering, leading to enhanced material functionality. Compared with traditional materials without such bio-inspired design elements, these composites offer a more holistic approach to hemostasis and wound healing [95].

**Figure 6:**
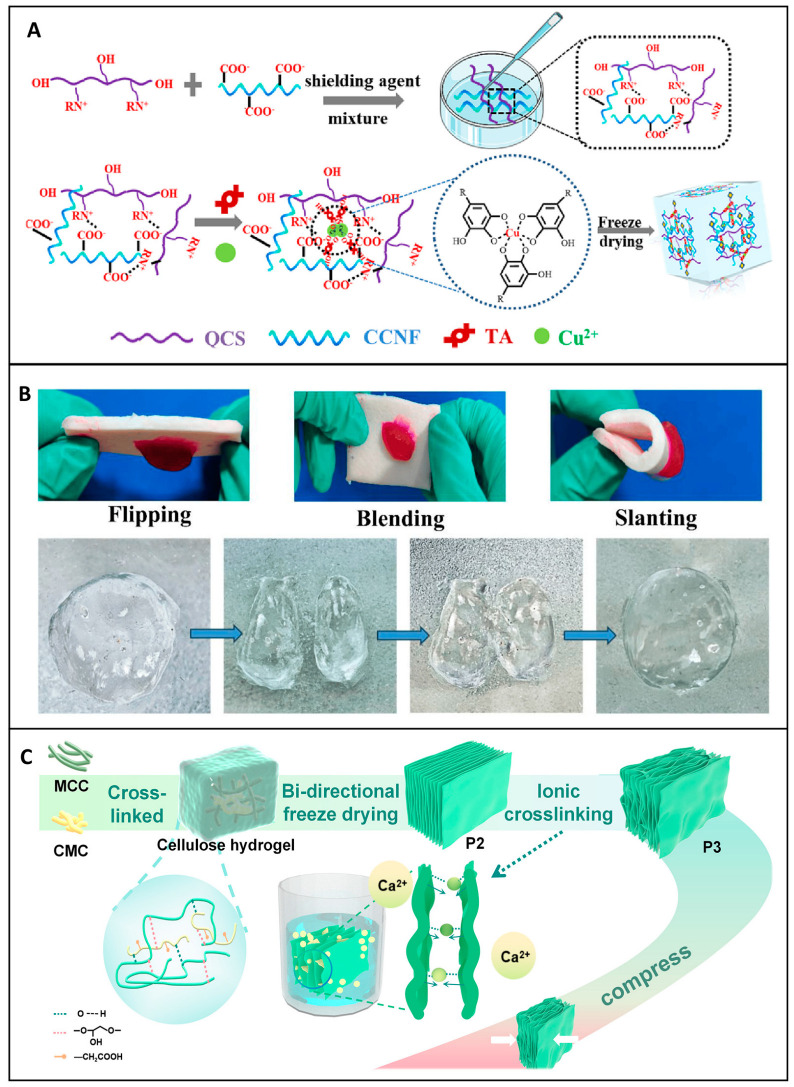
Advanced design strategies for cellulose-based hemostatic dressings. (**A**) Schematic of the fabrication of a microchanneled CQTC sponge dressing for enhanced hemostasis and wound healing. Reprinted with permission from Ref. [93]. Copyright 2023 Elsevier. (**B**) Illustration of the developed lysine-enriched self-gelatinizing powder composed of CMC-Ca synthesized via ion exchange and physically blended rapid gelation hemostasis and tissue regeneration. The arrows illustrate the step-by-step fabrication of the arch-like lamellar structure, from crosslinking to the final dressing for hemostasis. Reprinted from Ref. [96]. (**C**) Fabrication process of the arch-like lamellar cellulose sponge using bi-directional freezing followed by calcium ion crosslinking for noncomprehensive hemostasis. The arrows illustrate the step-by-step fabrication of the arch-like lamellar structure, from crosslinking to the final dressing for hemostasis. Adapted with permission from Ref. [97]. Copyright 2025 Elsevier.

Self-gelling powders represent another emerging direction of multifunctional wound care. Zhao et al. developed a novel lysine-enriched self-gelatinizing powder based on calcium carboxymethylcellulose (CMC-Ca), synthesized by ion exchange and physically blended with lysine (Lys) to enhance its functionality, as shown in Figure 6B. This formulation demonstrated water absorption, capable of retaining up to 30 times its weight in liquid, and formed a stable gel within seconds, key attributes for effective wound sealing and protection. In coagulation tests, the material achieved complete hemostasis within 3 min, highlighting its effectiveness in controlling bleeding. Importantly, the gel structure promotes adhesion to moist tissue surfaces, while its swelling capacity helps maintain a moist wound healing environment. This dual functionality for hemostasis and wound healing sets it apart from other single-purpose wound care products. Its unique microstructure and chemical composition enable it to be effective in wound dressing applications [96]. The continued exploration and innovation in the microstructural design of hemostatic materials, whether through porosity control, particle size optimization, incorporation of microchannels, or bio-inspired approaches, have led to significant improvements in their performance.

#### 4.1.3. Bioactive Coatings and Functional Enhancements

The integration of bioactive coatings has emerged as a crucial strategy to enhance the wound care capabilities of cellulose dressings. These coatings confer additional functionalities that are highly beneficial in promoting effective wound healing. Coatings containing tannic acid and Fe^3+^ ions or silver nanoparticles have been shown to significantly augment performance. The Fe^3+^-coated cellulose sponge, as investigated by Zhang et al., demonstrated rapid coagulation and potent bacteriostatic effects in animal models [98]. This is in contrast to uncoated cellulose dressings, which may lack such rapid hemostatic and antimicrobial properties. The addition of silver nanoparticles is a well-known approach to impact antimicrobial activity, and, when combined with cellulose, it provides an added layer of protection against wound infections [99]. Similarly, the incorporation of copper nanoparticles, as shown by Cheng et al., offers antimicrobial benefits while maintaining biocompatibility, as seen in Figure 5C. Their design of a microchannel structure based on nanofibrillated cellulose and quaterinated chitosan (QCS) and CCNF using tannic acid and a Cu^2+^ complex not only showed excellent fluid absorption and hemostasis capability but also highlights the potential of this particular coating strategy compared to other methods that may not achieve such a balance between functionality and biocompatibility [93].

Carbon nanofiber coatings present a unique advantage by creating superhydrophobic surfaces that repel blood and prevent microbial adhesion. Li et al. demonstrated that these superhydrophobic surfaces rapidly initiate coagulation and minimize bacterial colonization. In high-risk wound environments, such as those with a likelihood of microbial contamination, this property is of great significance [100]. In comparison to traditional wound dressings that may not have such a repellent surface, these carbon nanofiber-coated cellulose dressings offer enhanced protection and improved wound healing prospects.

Incorporating a hydrogel layer into cellulose dressings is another approach that has shown promise, particularly for enhancing absorption, conformability, and tissue adhesion. Building on these concepts, Cai et al. developed a compressible and expandable cellulose sponge designed specifically for non-compressible hemorrhage, a condition to manage in both civilian trauma and battlefield settings. Their innovative design features an arch-like lamellar structure fabricated by a bidirectional freezing technique followed by interlayer crosslinking by calcium ions (Figure 6C). They reported this architecture mimics natural load-bearing geometries, giving the dressing high mechanical resilience under compression while retaining its ability to expand rapidly upon hydration. Upon contact with body fluids, the sponge expands up to its initial size, filling deep and irregular wound cavities and exerting uniform pressure to tamponade bleeding. The interconnected porous microstructure and capillary effect facilitate rapid blood absorption, while the carboxyl-functionalized cellulose matrix and calcium ions actively stimulate platelet aggregation and accelerate the coagulation cascade through intrinsic and extrinsic pathways [97].

Mimicking natural structures, such as collagen aggregates, provides cellulose dressings with mechanical strength, hydrophilicity, and hemostatic performance similar to native tissues. Kacvinska et al. showed that mimetic structures promote platelet aggregation and fibrin formation. This is a significant improvement over dressings that do not possess these natural-like characteristics, as it allows for a more physiologically relevant approach to wound healing [101]. The incorporation of various bioactive coatings and mimicking natural structures into cellulose dressings offers a multitude of advantages. These strategies not only improve the hemostatic and antimicrobial properties but also improve the overall wound healing process.

#### 4.1.4. Mechanical Properties and Biocompatibility

The possession of adequate strength and flexibility is of utmost significance for dressings that are intended for mobile or irregular wound sites. These properties are not only for the practicality of the dressing during handling and application, but also for its long-term performance. Dressings with appropriate strength can withstand the mechanical stresses associated with movement and handling, preventing tearing or shifting. At the same time, flexibility, especially when accompanied by shape memory, allows the dressing to conform precisely to the contours of the wound, regardless of its complexity or mobility. For example, cryogels fabricated from TEMPO oxidized bacterial cellulose have been demonstrated to have high absorbency and tensile strength [102]. This combination of properties ensures durability of the dressing and reduces the need for frequent replacement but also makes it easier for healthcare providers to handle.

Similarly, alkylated chitosan sponges and aerogels exhibit robust structures and shape recovery behavior. Their ability to accommodate high absorption requirements is particularly advantageous in the context of non-compressible wounds, such as those that are deep and irregular [103,104]. This is because they can effectively manage the exudate while facilitating hemostasis, a function that may not be as effectively achieved by other types of dressings. Compared to dressings with less effective absorption capabilities, these materials can maintain a microenvironment more conducive to wound healing by preventing the accumulation of excess fluid, which could otherwise impede the healing process or increase the risk of infection.

Biocompatibility and the minimization of immune response are equally, if not more, essential aspects of an effective wound dressing. A biocompatible dressing is one that can interact with biological tissues at the wound site without triggering harmful immune reactions. This is essential to ensure that the dressing promotes healing naturally and unhindered, without introducing additional complications [105]. For instance, some materials can cause inflammation or allergic reactions, which can delay wound healing and cause discomfort to the patient. By contrast, a dressing with excellent biocompatibility, such as those designed with advanced cellulose-based composites, can integrate seamlessly into the wound environment, allowing the body’s natural healing mechanisms to function optimally [106]. This is achieved through careful material selection and surface modifications that reduce the likelihood of immune recognition and subsequent adverse responses. Future research could focus on further optimizing the balance between strength, flexibility, biocompatibility, and other functional properties of wound dressings to meet the diverse and evolving needs of patients and healthcare providers. Additionally, comparative studies between different types of dressings could provide more detailed information on their relative performance in various clinical scenarios, guiding more informed decisions in wound care management.

### 4.2. Fabrication Design Strategies for Cellulose-Based Wound Dressings

Creating effective cellulose-based wound dressings requires a balance of material selection, chemical modification, microstructural engineering, and manufacturing techniques. This integrated approach combines the natural properties of cellulose with advanced design strategies to promote effective wound healing.

#### 4.2.1. Material Selection and Chemical Modification

The choice of the material plays a pivotal role in dictating the characteristics and effectiveness of a wound dressing. Bacterial cellulose (BC) has emerged as a favored base material for wound dressings owing to its remarkable biocompatibility, outstanding water retention capabilities, and high degree of flexibility. These inherent properties allow BC to promote a moist wound environment at the wound site, which is conducive to the healing process and serves to prevent the dressing from adhering to the wound bed, a common problem with many traditional dressings [49].

To further increase its functionality, BC can be combined with additional polymers like chitosan or polyvinyl alcohol (PVA), as well as bioactive agents such as silver nanoparticles. This combination imparts antimicrobial and healing-promoting traits. For example, when BC is modified with quaternized chitosan, it exhibits an enhanced swelling capacity and improved antimicrobial efficacy. This makes it particularly advantageous in the treatment of infected or exuding wounds, where traditional dressings may not be as effective in managing moisture balance and preventing bacterial proliferation [107].

Chemical functionalization represents another avenue for enhancing the performance of BC. Through techniques such as hydrothermal synthesis, sol-gel methods, and gas-liquid interface reactions, bioactive molecules and antimicrobial agents can be incorporated. These modifications bolster the dressing’s ability to control infection and also improve biocompatibility. This, in turn, promotes more favorable cellular interactions, which are of utmost importance in the wound healing process. Moreover, the functional groups introduced via these methods can enhance the adhesion of bioactive agents and optimize drug release profiles, ensuring a controlled and sustained therapeutic effect.

**Figure 7:**
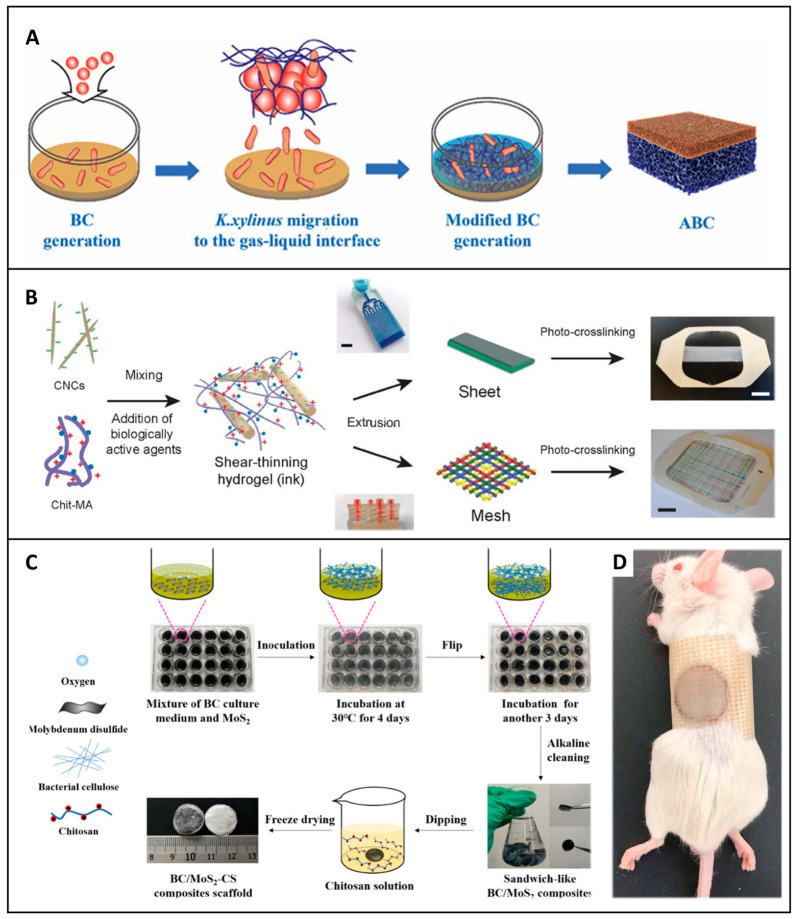
Fabrication strategies for multifunctional cellulose-based wound dressings. (**A**) Asymmetric bacterial cellulose wound dressing prepared by gas-liquid assembly. Adapted with permission from Ref. [108]. Copyright 2023 John Wiley and Sons. (**B**) 3D-printed wound dressing made from cellulose nanocrystal-based hydrogel ink by microfluidic extrusion and UV curing. Reprinted with permission from Ref. [111]. Copyright 2021 merican Chemical Society. (**C**) BC/MoS_2_-chitosan nanocomposite wound dressing with a sandwich structure manufactured by in situ growth. Silver nanowire-BC wound dressing prepared by freeze-drying. Reprinted with permission from Ref. [115]. Copyright 2021 merican Chemical Society. (**D**) Wound healing after covering with silver-nanowire-BC based wound dressings. Adapted with permission from Ref. [121]. Copyright 2020 Elsevier.

Feng et al. developed an asymmetric BC wound dressing using the gas-liquid method incorporating bioactive agents, as illustrated in Figure 7A. The innovative dressing not only inhibits the growth of bacteria on infected wounds but also regulates the release of curcumin. These functionalities result in a reduction in inflammation and actively promote the production of epithelium, blood vessels, and collagen, a combination of benefits that distinguishes it from many other BC-based dressings [108]. The selection and modification of materials such as BC offer important opportunities to develop more effective wound dressings by improving their structural, biological, and therapeutic functionalities.

#### 4.2.2. Microstructure Engineering and Fabrication Techniques

To optimize the healing process, cellulose-based dressings necessitate a well-optimized microstructure. This microstructure should be designed to proficiently support cell attachment, facilitate efficient wound drainage, and effectively control infection. Microstructure engineering at the macro and nano scales empowers precise control over porosity, surface topography, and fiber alignment. It increases essential properties such as flexibility, breathability, and water retention. These enhanced characteristics render the dressing more adaptable to irregular wound shapes, thereby and significantly improving patient comfort. A diverse array of manufacturing techniques has been developed to enable the precise design of these microstructures. Among them, 3D printing stands out, as it allows for the controlled fabrication of cellulose-based dressings, achieved through a layer-by-layer deposition process, which offers the unique advantage of customizing mechanical properties and swelling behavior.

In the realm of cellulose-based composite wound dressings, researchers have delved into the integration of cellulose with other biomaterials to fabricate customized structures tailored for hemostasis and wound healing. For example, studies have been conducted on the 3D fabrication of cellulose composite as an adhesive wound dressing to expedite the wound healing process [109]. In one instance, anisotropically printed poly(N-isopropylacrylamide) ink was incorporated onto cellulose nanofiber wound dressing for direct wound ink writing. The addition of nanocellulose was found to substantially influence the printing parameters, which in turn strongly determined the resulting degree of orientation. The results obtained paved the way for the creation of complex three-dimensional structures with programmable actuation for hydrogel wound dressing, a significant advancement over traditional dressings that lack such precise control over structure and functionality [110].

When it comes to incorporating antimicrobial or growth-promoting agents into 3D-printed structures, it becomes possible to engineer wound dressings with targeted drug release profiles specifically designed to address specialized wound types. For instance, as depicted in Figure 7B, a study demonstrated the use of 3D printed cellulose nanocrystal hydrogel containing silver nanoparticles and growth factors. This innovative dressing showed improved granulation tissue formation and vascular density tailored to meet specific healing requirements. In contrast to conventional dressings that may only offer basic wound coverage, this 3D printed hydrogel dressing provides a more targeted approach to wound healing, potentially speeding up the recovery process [111].

Electrospinning is another technique that has gained prominence in the production of cellulose-based dressings. It produces nanofibrous mats with high surface area and porosity, which are highly conducive to cell adhesion and proliferation. This method allows embedding antimicrobial agents, such as silver nanoparticles, or polymers, such as chitosan. These incorporated agents not only provide continuous protection against infections but also actively promote cell growth. Electrospun cellulose nanofibers possess the remarkable ability to conform well to complex wound shapes, enhancing both flexibility and comfort [112]. For example, electrospun cellulose-based nanofibers containing citric acid quantum dots (CA-QDs) have demonstrated promising antibacterial efficacy against common wound pathogens, highlighting the potential for customized, infection-resistant wound dressings using electrospinning technology [113]. Similarly, the incorporation in a chitosan/poly (ethylene oxide) blend has been shown to enhance antifungal activity while maintaining antibacterial efficacy, a feat that may not be achievable with simpler wound dressing formulations [114]. These findings suggest that electrospun cellulose has the potential to serve as a highly effective localized drug delivery system for wound care applications, offering a more targeted and efficient approach to wound treatment compared to traditional topical treatments.

Layer-by-layer assembly or the creation of multilayered structures enables the precise control of multilayer dressings. This is accomplished by sequentially adding functional materials such as antimicrobials, growth factors, or hydrogels. This technique offers remarkable flexibility in material combinations and allows for the creation of bioactive surfaces that can regulate the release of therapeutic agents. As a result, it is ideally suited for the management of acute and chronic wounds. A notable example is the sandwich-like structure, where cellulose nanofibers are used to create a layered membrane in situ, as described in Figure 7C. The layered composite structure offers the distinctive advantage of being adaptable to different stages of the infected wound. Initially, it ensures effective hemostasis, then promotes cell growth and healing. This is a significant improvement over single-layer dressings that may not be able to meet the evolving needs of a healing wound [115]. A similar study by Wang et al. designed a cellulose-based wound dressing sponge with sandwich-like layers that were combined with silk fibroin, graphene-based polyvinyl alcohol, and chitosan-konjac glucomannan. It was reported that the dressing could improve wound healing with superior healing characteristics, with wounds shrinking by two times compared with a commercial dressing. These results clearly indicate that the unidirectional wicking dressing has the potential to become the next generation of clinical wound dressings, outperforming existing options in terms of efficacy and functionality [116].

Freeze-drying, also known as lyophilization, is widely employed in the manufacture of porous cellulose-based wound dressings. Its popularity stems from its ability to create highly porous structures that excel in fluid absorption and maintain a moist healing environment. Freeze-drying is particularly well-suited for the creation of porous structures suitable for highly exuding wounds and advanced scaffolds for wound care, including composites that integrate multiple biomaterials for enhanced functionality [117]. Cellulose-based sponges manufactured through freeze-drying can be infused with nanoparticles to confer hemostatic and antimicrobial benefits. For example, studies such as that of Bi et al. showed that these dressing possess superior hemostasis, antibacterial properties, and oxidative stability, proving effective in skin repair [118]. Similarly, Wei et al. developed quaternized chitosan/cellulose sponges that exhibit excellent hemostatic, antibacterial, and blood-absorbing qualities ideal for battlefield or hospital dressings. These sponges outperform many traditional dressings in terms of multifunctionality and effectiveness in addressing severe bleeding and infection risks [119].

Cellulose aerogel wound-based dressings with the ice-template method are formed by freeze-drying to achieve high porosity. Zheng et al., for instance, created ultra-long hydroxyapatite nanowires and an ultra-long hydroxyapatite nanowire aerogel dressing that rapidly absorbs blood, promoting wound coagulation and healing. The dressing, with its super hydrophilicity and cytocompatibility, has been shown to be effective in animal models, suggesting the potential for rapid wound treatment [120].

Cellulose-based hydrogels formed by freeze-drying are biocompatible, biodegradable, and have high fluid absorption capacity. They are also stimuli-responsive, meaning they can respond to certain stimuli, usually combined with PVA or loaded with nanoparticles. Studies such as that of Yi et al. highlight a BC hydrogel dressing with polydopamine and PVA designed to gradually release antibiotics such as doxycycline. The dressing outperformed commercial alternatives in a mouse wound model, showing excellent wound closure, mechanical strength, and infection control. This is an important achievement, as it demonstrates the potential of cellulose-based hydrogel dressings to surpass traditional wound dressings in terms of overall performance and therapeutic efficacy [68]. Similarly, coatings with bioactive agents, such as tannic acid, silver nanoparticles, or antibiotics, enhance antibacterial properties. For example, BC-coated Ag+ shows excellent in vivo healing ability and biocompatibility, as seen in Figure 7D, a combination of properties that is highly desirable in a wound dressing [121]. In Shaaban et al., green-synthesized silver nanoparticles were incorporated into bacterial cellulose membranes to achieve high antibacterial activity, targeting infections caused by pathogens [122].

These approaches vividly highlight innovative uses of cellulose and its derivatives in wound dressings, with a strong emphasis on enhanced hemostatic properties, antimicrobial protection, and tissue regeneration to improve wound care outcomes. By leveraging these advanced manufacturing techniques and material combinations, the goal is to significantly improve wound care outcomes, providing more effective and efficient treatment options than what has been available in the past. Future research could focus on comparing the long-term effects of these different cellulose-based dressing formulations on wound healing and scar formation, as well as exploring their potential applications in more complex wound scenarios, such as those involving patients with compromised immune systems or chronic comorbidities.

## 5. Application of Cellulose-Based Dressings for Hemostasis and Wound Healing

Recent advances in cellulose-based dressings reveal significant potential for hemostasis and wound healing, as demonstrated through extensive research and preclinical studies. These dressings exploit different forms of cellulose, including plant cellulose, bacterial cellulose, nanostructured cellulose, and chemically modified cellulose, each of which provides unique properties to control bleeding, enhance tissue repair, and ensure biocompatibility.

### 5.1. Hemostatic Applications

Cellulose-based dressings have firmly established themselves as highly versatile and invaluable assets in the evolution of advanced hemostatic dressings. Their fundamental mechanism of action in bleeding control is multifaceted. Due to their ability to promote clot formation, they actively engage in the physiological process of hemostasis. This occurs through interactions with platelets and clotting factors, effectively jumpstarting the coagulation cascade. Simultaneously, they are capable of activating specific coagulation pathways, further enhancing the body’s natural response to injury. Moreover, by providing a physical barrier at the wound site, they prevent excessive blood loss and protect the wound from external contaminants. Crucially, these dressings maintain both biocompatibility and hemocompatibility, meaning they can interface with biological tissues and blood components without eliciting harmful immune responses or interfering with normal blood function. This unique combination of properties makes them indispensable in the domains of trauma care and wound management [19].

**Figure 8:**
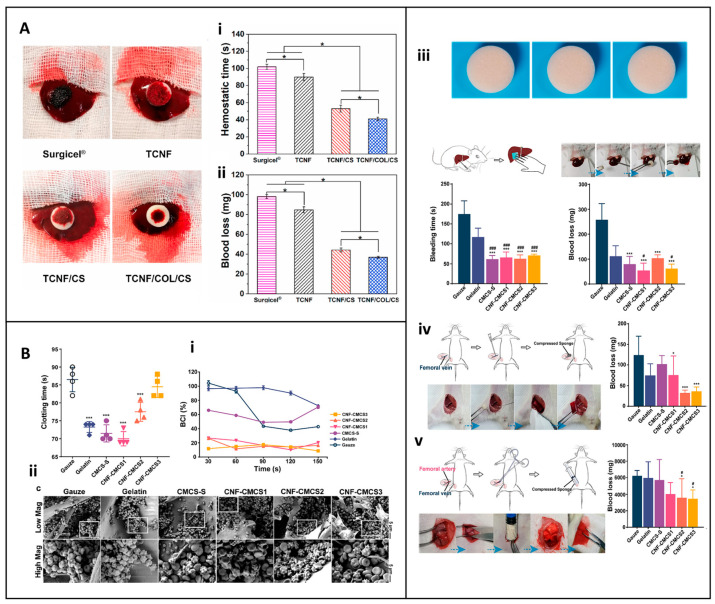
Hemostatic efficacy and applications of nanocellulose-based aerogel and sponge dressings. (**A**) In vivo evaluation of TCNF/COL/CS aerogel dressing in a rat liver injury model: (**i**) Quantification of hemostatic time, (**ii**) Blood loss analysis. Reprinted from Ref. [89]. (**B**) Hemostatic performance of CNF-CMCS sponge dressing; (**i**) Whole blood clotting time, (**ii**) Blood coagulation index (BCI), (**iii**) SEM images of erythrocyte and platelet adhesion, (**iv**) In vivo results in mouse and rat liver trauma models, (**v**) Statistical comparison with conventional dressings in femoral puncture and deep wound models. **i**,**ii**,**iv**,**v** are reprinted with permission from Ref. [24]. Copyright 2022 Elsevier. **iii** is reprinted from Ref. [89]. * *p* < 0.05, *** *p* < 0.001, # *p* < 0.05, ### *p* < 0.001; Data are presented as mean ± SD.

In recent times, there has been a flurry of activity in the research and development of cellulose-based dressings, leading to a significant expansion of their functional applications. These now encompass not only enhanced hemostatic efficacy but also the incorporation of antimicrobial properties and the promotion of tissue regeneration. For example, Lu et al. developed a hemostatic aerogel combining TEMPO oxidized cellulose nanofiber (TCNF), collagen, and chitosan (TCNF/COL/CS) dressing. This material has a porous interconnected structure that enhances blood absorption, platelet adhesion, and clot formation as described in Figure 8A. In vivo studies demonstrated that the procoagulant properties of the dressing surpassed those of traditional materials such as Surgicel^®^, reducing blood loss by 62.2% and improving hemostatic efficacy by 59.8%. In addition, its antibacterial properties against *E. coli* (96.8%) and *S. aureus* (95.4%) and biodegradability make it a promising candidate for internal bleeding control, especially when traditional materials are inadequate [89].

Similarly, Wang et al. devised an oxidized bacterial cellulose (OBC) aerogel combined with platelet extracellular vesicles (pVEs). This innovative dressing exhibited self-expanding properties, which enabled it to adapt and form a reliable physical barrier even in the case of irregularly shaped wounds. By concentrating platelets and clotting factors, it accelerated the clotting process, as evidenced by a substantial reduction in blood loss and bleeding time in rat liver and tail injury models [104]. This represents a significant advancement over traditional wound dressings, which may not have the ability to actively enhance the clotting process in such a targeted manner.

Additionally, Zhou et al. developed an innovative expandable hemostatic sponge composed of carboxymethyl chitosan (CMCS) and cellulose nanofibers (CNFs), synthesized by CO–NH crosslinking. The resulting CNF-CMCS dressing exhibits rapid expansion upon contact with blood, enabling it to effectively absorb fluids and exert mechanical pressure on the wound mechanisms that facilitate rapid coagulation, as illustrated in Figure 8B. In vitro tests revealed its potent procoagulant effects, with the sponge surface promoting platelet adhesion and activation. SEM analysis showed clusters of red blood cells and activated platelets with extended pseudopodia, indicating triggered coagulation cascades (Figure 8(Bi,Bii)). In vivo studies, including liver puncture and deep penetrating wound models, demonstrated a significant reduction in bleeding time and blood loss compared to gauze and gelatin controls (Figure 8(Biii–Bv)) [24]. These results position the CNF-CMCS sponge as a promising hemostatic agent for the management of severe and uncontrolled hemorrhage, particularly in penetrating wounds.

**Figure 9:**
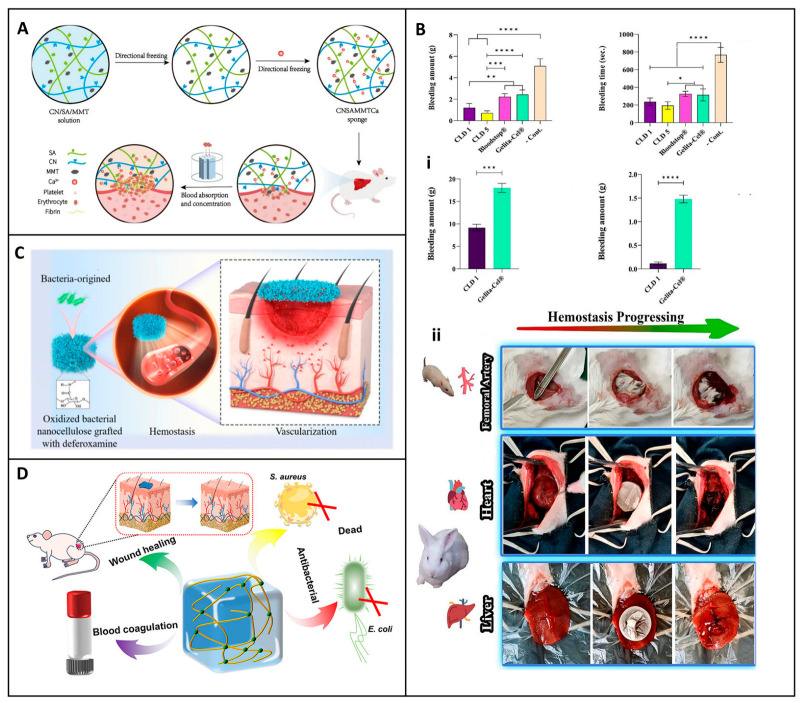
Synthesis and evaluation of hemostatic functionality of nanocellulose-based sponge and hydrogel wound dressings. (**A**) Schematic illustration of hemostatic mechanism of CNSAMMTC sponge dressing. Reprinted with permission from Ref. [123]. Copyright 2024 Elsevier. (**B**) In vivo hemostatic performance of multifunctional nanocellulose sponge dressing: (**i**) Blood loss in a rat femoral artery model, (**ii**) Bleeding time reduction in rabbit heart and rat liver models. Reprinted with permission from Ref. [124]. Copyright 2024 Elsevier. (**C**) Preparation of OBNC and OBNC-DFO nanocomposites from bacterial nanocellulose (BNC) and their dual function in promoting hemostasis and vascularization. Reprinted with permission from Ref. [124]. (**D**) Oxidation process of cellulose to dialdehyde cellulose (DAC) hydrogel and schematic of its antibacterial interactions and wound healing effects. Reprinted from Ref. [125]. * *p* < 0.05, ** *p* < 0.01, *** *p* < 0.001, **** *p* < 0.0001. Data are presented as mean ± SD.

Liu et al. took a different approach by designing a water-triggered shape memory sponge using carboxylated cellulose nanofibers (CN), sodium alginate (SA), and montmorillonite (MMT). This sponge was able to activate the coagulation cascade, a feat that many conventional wound dressings achieve. It also demonstrated excellent hemocompatibility, as summarized in a chart in Figure 9A. In animal models, it effectively reduced blood loss, highlighting its potential for rapid coagulation and cytocompatibility [123]. The combination of these properties makes it a promising candidate for clinical applications, especially in scenarios where rapid hemostasis is of the essence. The multifunctionality of cellulose-based hemostats was exemplified by Mahmoodzadeh et al., who established an innovative nanoclay-modified cellulose sponge dressing (CLD) designed to address the clinical challenges of controlling severe, non-compressible bleeding (Figure 9B). This dressing demonstrated superior adhesive strength (~405 kPa) and rapid blood absorption capacity, which are essential for achieving rapid hemostasis in high-pressure bleeding (Figure 9(Bi)). In vivo evaluation in a rat femoral artery bleeding model provided compelling evidence of its hemostatic efficacy. Without the application of external pressure, CLD1 and CLD5 samples significantly reduced blood loss to 1.2 g and 0.71 g, respectively, compared to 2.22 g and 2.43 g recorded for the commercial controls Bloodstop^®^ and Gelita-Cel^®^. The dressing’s efficacy was also verified in high-pressure bleeding models in rabbit heart and liver injury, conditions that challenge most conventional hemostatic agents due to tissue movement and increased blood flow (Figure 9(Bii)). In the rabbit heart model, the CLD1 sample was applied with minimal pressure, adhering effectively even under constant cardiac movement [124]. Bian et al. extended this concept by developing an oxidized bacterial nanocellulose (OBNC) sponge incorporating desferrioxamine (DFO). The dressing not only accelerated coagulation but also promoted vascularization through sustained release of DFO, thus addressing the two critical aspects of wound healing: hemostasis and tissue repair, as described in a diagram chat illustration in Figure 9C. This dual-action functionality distinguishes it from many other wound dressings that may focus on only one aspect of wound management [125].

Nanofiber-based dressings have also emerged as a promising area of research. Li et al. presented a quaternized chitosan/oxidized bacterial cellulose cryogel dressing with shape memory properties. This dressing effectively absorbed blood, activated coagulation pathways, and inhibited bacterial growth. The mechanical resilience and antimicrobial activity make it particularly suitable for managing emergent bleeding scenarios, where both hemostasis and infection prevention are crucial [62]. Yin et al. further improved the hemostatic efficacy with a sponge constructed from carboxylated nanocellulose and montmorillonite. This sponge dressing synergistically concentrates blood cells and activates the coagulation cascade, leading to rapid hemostasis in liver injury and tail amputation models, providing a practical solution for trauma management [65].

Besides aerogels, sponges, and cryogels, hydrogels have attracted considerable attention due to their adaptability in conforming to irregular wound surfaces, which holds great promise. Yi et al. developed a self-healing hydrogel dressing by introducing borate ester linkage bonds into tannic-acid-modified bacterial cellulose. The hydrogel dressing exhibited excellent mechanical properties, adhesion, and hemostatic ability, reducing blood loss by approximately 80% in controlled models [126]. Dong et al. developed a multifunctional cellulose-based hydrogel wound dressing by integrating 2-hydroxypropyl-β-cyclodextrin (HP-β-CD), iodine, and L-glutamine, designed to provide rapid hemostasis, long-term bactericidal activity, and improved wound healing, as illustrated in Figure 9D. The preparation involved forming an inclusion complex of iodine and HP-β-CD using a saturated aqueous solution method. Coagulation studies demonstrated the hydrogel’s ability to absorb four times its weight in blood and significantly reduce blood loss. In hemostasis tests, blood loss with the HP-β-CD/iodine hydrogel surpassed that of traditional gauze. These results suggest that chemical modification of cellulose can significantly improve its coagulative function. The dressing further accelerated wound closure and promoted tissue regeneration in vivo in a full-thickness murine scald model [127].

**Figure 10:**
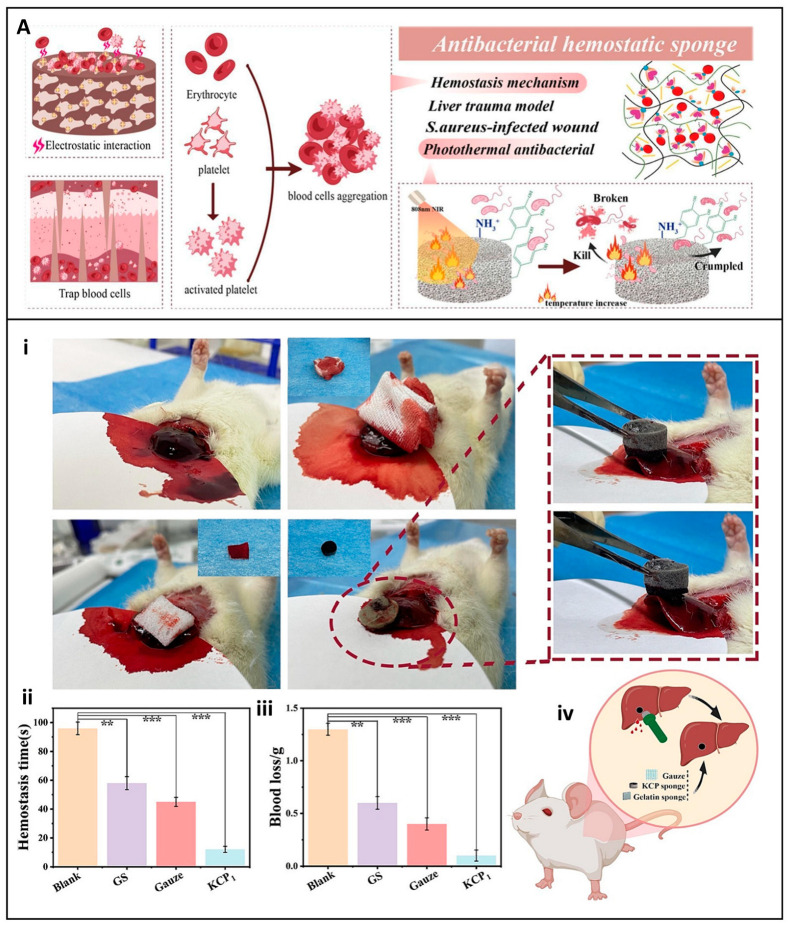
Evaluation of a multifunctional tunicate cellulose-based sponge dressing for improved hemostasis and wound healing. (**A**). Schematic of the synthesis and multifunctional mechanism of the KCP sponge dressing, composed of oxidized konjac glucomannan, chitosan, tunicate nanocellulose, and polydopamine, manufactured by two-step crosslinking and NIR photothermal activation. Adapted with permission from Ref. [128]. Copyright 2023 Elsevier. (**i**,**ii**). In vivo performance of the TCNC-based composite sponge compared with commercial gauze and sponge in rat liver trauma models. (**A**) (**i**–**iv**) Quantitative evaluation of hemostasis time, blood loss, and schematic representation of hemostatic efficacy in the rat liver trauma model. Reprinted with permission from Ref. [128]. Copyright 2023 Elsevier. ** *p* < 0.01, *** *p* < 0.001; Data are presented as mean ± SD.

Moreover, the integration of hemostatic and antimicrobial properties has been a subject of extensive study. In this context, Han et al. reported a multifunctional polysaccharide-based sponge dressing with integrated hemostatic and photothermal antimicrobial properties. The sponge was designed using a two-step crosslinking method, as illustrated in Figure 10A. Initially, oxidized konjac glucomannan (OKGM) and chitosan (CS) were crosslinked to form a dynamic covalent bond network, while tunicate-derived nanocellulose (TCNC) was uniformly incorporated to construct a stable three-dimensional fibrous structure. In the secondary crosslinking step, polydopamine nanoparticles (PDA NPs) were introduced, contributing to photothermal functionality and antioxidant capacity. In rat liver trauma models, the composite sponge achieved rapid hemostasis in 12 ± 2.17 s with minimal blood loss (0.1 ± 0.052 g), significantly outperforming gauze (45 ± 3.15 s, 0.4 ± 0.058 g) and gelatin sponges (58 ± 4.56 s, 0.6 ± 0.060 g) (Figure 10(Ai–Aiv)). In addition to its hemostatic efficacy, the sponge demonstrated excellent antimicrobial performance and furthermore accelerated healing by preventing infection and promoting tissue regeneration [128]. Fan et al. designed a chitosan-grafted cellulose sponge using a gas foaming method, which rapidly sealed bleeding wounds and significantly reduced the hemostasis time in animal experiments while exhibiting bacterial inhibition [129].

The dual-purpose potential of these materials is further highlighted in the study of Wang, who designed oxidized sodium carboxymethylcellulose composite hydrogel dressing. This checkerboard-patterned dressing with the hemostatic and wound healing capabilities conferred antibacterial properties, making the dressing suitable for the management of infected wounds [130]. Cao introduced an injectable cryogel based on aldehyde bacterial cellulose and polydopamine with photothermal antibacterial properties; the dressing demonstrated excellent blood absorption and enhanced hemostatic efficacy. The effective killing of bacteria offered versatility for the care of deep and irregular wounds [131]. Similarly, Shi et al. developed a light-responsive cellulose nanofibril (CNF)-based hydrogel dressing in situ for the healing of wounds infected with drug-resistant bacteria. The dressing demonstrated exceptional hemostatic performance and also provided robust antibacterial activity against *E. coli*, *S. aureus*, and MRSA, and facilitated wound healing in MRSA-infected models [132].

Other innovations in electrospun nanocellulose wound dressings have shown outstanding potential in the application of hemostatic materials. Lui et al. developed a hydrophobic electrospun nanocellulose wound dressing by doping N-alkylated chitosan into chitosan/polyethylene oxide nanofibers. The optimized formulation (CPN31) significantly reduced clotting time, minimized blood loss, and prevented secondary hemorrhage. It demonstrated excellent hemostatic properties, biocompatibility, blood compatibility, and fibroblast proliferation, making it a promising material for hemorrhage control [133]. These studies collectively demonstrate the remarkable versatility of cellulose-based hemostatic materials. Taken together, these studies vividly illustrate the remarkable versatility of cellulose-based hemostatic materials. From sponges and aerogels to cryogels and hydrogels, the continuous innovations in material design and functionalization are propelling the field forward. By integrating rapid hemostasis, biocompatibility, antimicrobial activity, and tissue repair capabilities, cellulose-based dressings are poised to revolutionize trauma care and wound management. Future research could focus on comparing the performance of these different cellulose-based dressings in more complex clinical scenarios, such as those involving patients with comorbidities or in extreme environmental conditions. Additionally, studies could explore the long-term effects of these dressings on tissue remodeling and scar formation, which would provide more comprehensive information for their clinical application.

### 5.2. Wound Healing Applications

Cellulose dressings have emerged as a very promising area in the field of wound healing, exhibiting remarkable hemostatic efficacy and a multitude of other beneficial properties. Their multifunctionality is a key aspect that distinguishes them in the realm of wound care. They possess antibacterial activity, essential for preventing wound infections, and promote a sterile environment conducive to healing. The biocompatibility of these dressings is of the greatest importance, as it ensures that they can be safely integrated into biological tissues without eliciting adverse immune responses. Additionally, their resistance to cytotoxicity further enhances their suitability for wound treatment. By modifying cellulose structure or incorporating various additives such as antibacterial agents, these composite dressings can effectively prevent infections and accelerate the wound healing process [134]. For instance, modified bacterial cellulose (BC)-based materials have shown great potential in enhancing cytocompatibility. They support the proliferation of fibroblasts and endothelial cells, which are essential for tissue regeneration. This is in contrast to some traditional wound dressings that may not provide as favorable an environment for cell growth [135].

**Figure 11:**
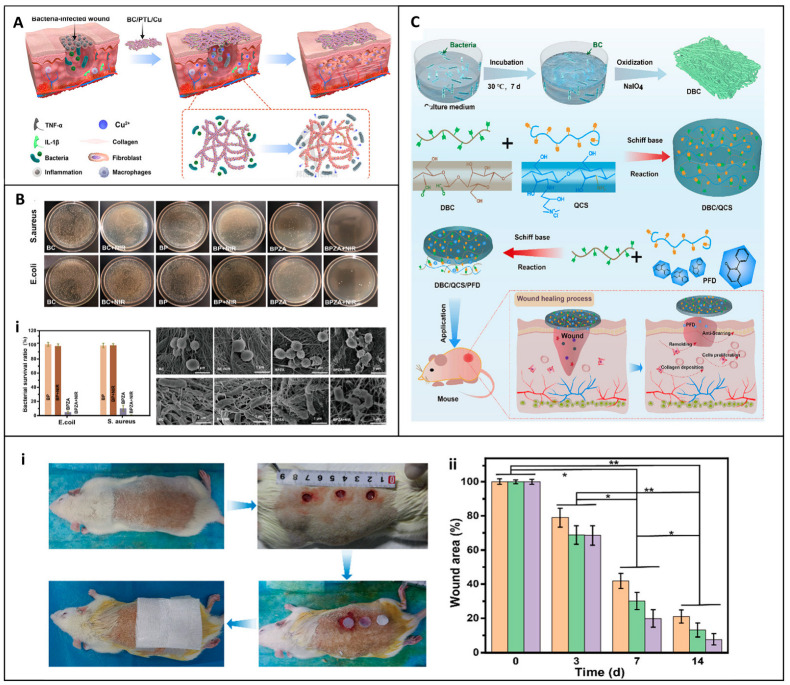
Selective images of multifunctional BC-based dressings with antibacterial and regenerative capacity for wound healing applications. (**A**) Fabrication scheme and mechanism of action of the BC/PTL/Cu dressing. Adapted with permission from Ref. [136]. (**B**) Photographs of bacterial colonies of *E. coli* and *S. aureus* treated with BPZA dressings; (**i**) Bacterial survival rate and SEM of bacterial morphology. Adapted with permission from Ref. [107]. (**C**) Synthesis of dehydrated BC hydrogel crosslinked with quaternized chitosan via Schiff base reactions DBC/QCS. (**i**,**ii**) In vivo wound healing performance and their relatives wound closure rates. Adapted with permission from Ref. [137]. * *p* < 0.05, ** *p* < 0.01; Data are presented as mean ± SD.

When it comes to the incorporation of antibacterial agents, several studies have demonstrated their efficacy. He et al. developed a novel bacterial cellulose (BC) dressing modified with Cu^2+^-loaded phase-transition lysozyme (PTL) nanofilms on BC matrices (BC/PTL/Cu), forming a bioactive interface with broad therapeutic potential (Figure 11A). Through electrostatic coordination, Cu^2^⁺ ions were stably incorporated into the PTL layers, enabling sustained antimicrobial action against various pathogens, including *S. aureus*, *E. coli*, *B. subtilis*, and *P. aeruginosa*. In a rat full-thickness infected wound model, the dressing promoted tissue regeneration, maintained optimal moisture and oxygen exchange, and suppressed local inflammation. These combined effects facilitated epithelial regeneration, collagen deposition, and angiogenesis, establishing a comprehensive approach to wound healing [136].

Similarly, Deng et al. developed a BC-based hydrogel wound dressing incorporating polydopamine (PDA) and silver-loaded zeolitic imidazolate framework-8 (ZIF-8) nanoparticles. This hybrid system utilized both chemical and photothermal antibacterial strategies (Figure 11B). The acidic wound microenvironment triggered a controlled release of Zn^2+^ and Ag⁺ ions, while NIR-induced photothermal conversion by PDA elevated local temperatures to inhibit bacterial enzymatic functions. Remarkably, bacterial survival rates fell below 1% under near-infrared irradiation. SEM analysis revealed significant membrane disruption, confirming the potent bactericidal effects (Figure 10(Ai–Aiii)). Moreover, this approach stimulated vascularization and tissue repair, positioning it as a powerful strategy for managing infected wounds through synergistic antibacterial mechanisms [107].

Further advancements in BC-based materials include addressing limitations such as high water content and structural instability during storage. Deng et al. addressed these challenges by modifying BC with quaternized chitosan (QCS), producing a novel dehydrated hydrogel, termed DBC/QCS, with exceptional swelling capacity and rehydration functionality suitable for clinical use (Figure 11C). The introduction of QCS, a bio-based polyelectrolyte containing positively charged amine groups, into dialdehyde bacterial cellulose (DBC) via Schiff base reactions resulted in a crosslinked hydrogel network capable of repeated dehydration and rehydration cycles without compromising its structure (Figure 11(Ci,Cii)). This material demonstrated excellent rehydration properties, swelling over 1000% to regain its hydrogel form while maintaining its mechanical integrity. The presence of positively charged QCS conferred strong antibacterial efficacy to the matrix, achieving >80% bacterial kill against *E. coli* and *S. aureus* through electrostatic membrane disruption. Biocompatibility tests confirmed fibroblast growth, and in vivo evaluations showed improved wound healing, enhanced collagen deposition, and reduced scarring, particularly in combination with pirfenidone (PFD) (Figure 11(Ci,Cii)). Altogether, BC-based systems exemplify the evolution of wound dressings from passive barriers to dynamic therapeutic platforms. Whether through sustained metal ion release, photothermal synergy, or modular rehydration mechanisms, each formulation offers distinct advantages suited to complex wound environments [137].

**Figure 12:**
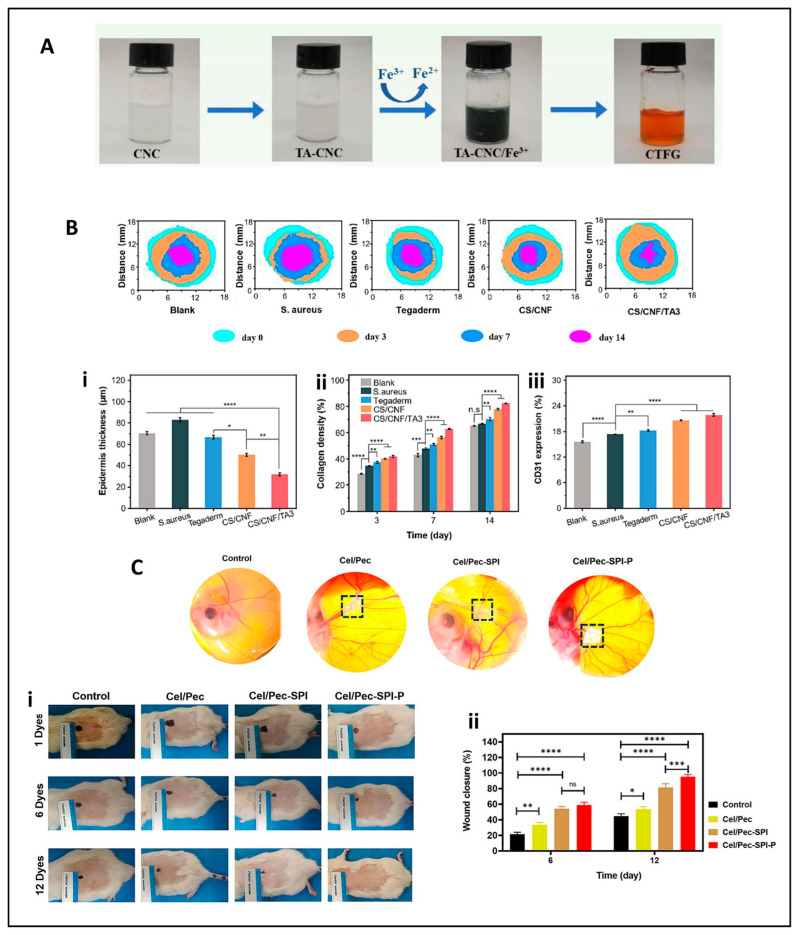
Advanced multifunctional nanocellulose-based dressings for improved wound healing application. (**A**) Illustration design of CTFG@S hydrogel of TA-CNC/Fe^3+^ hydrogel combined with silk fibrin (CTFG@S) for potent infected wound. Adapted with permission from Ref. [138]. (**B**) The performance of the CS/CNF/TA hydrogel dressing shows strong antibacterial activity and almost complete wound closure; closure of CS/CNF/TA-treated wounds, collagen deposition, and neovascularization of treated wounds. (**i**) Epithelial thickness analysis; (**ii**) collagen deposition; (**iii**) neovascularization. Adapted with permission from Ref. [139]. Copyright 2024 Elsevier. (**C**) The dashed area indicated new vessels formed at dressing location; (**i**) Epidermal regeneration with the Cel/Pec-SPI-P compared to in-complete healing in other groups; (**ii**) wound closure rate. Adapted with permission from Ref. [140]. Copyright 2024 Elsevier. * *p* < 0.05, ** *p* < 0.01, *** *p* < 0.001, **** *p* < 0.0001; Data are presented as mean ± SD.

Beyond bacterial cellulose, other forms of cellulose, including plant-derived nanocellulose and cellulose nanocrystals, demonstrated significant potential in wound care. These materials are often enhanced with tannic acid coatings, chitosan, or embedded nanoparticles to achieve robust antibacterial, UV-resistant, and enhanced mechanical properties to combat moisture imbalance, delayed tissue regeneration, and infections in the wound. For instance, Yang et al. presented an electrospun silk fibroin-reinforced TA-CNC/Fe^3+^ hydrogel (CTFG@S), producing a self-adhesive, UV-resistant, and fatigue-resistant bilayer structure. The hydrogel’s rapid gelation (~6 s), remarkable flexibility (800% strain), and tolerance to cyclic compression demonstrate a significant advance in mechanical robustness. Notably, the composite incorporates moisture-sensing capabilities, positioning it as a potential platform for smart wound dressings, providing real-time feedback while preserving skin compliance and stability. These features directly address common mechanical limitations of conventional hydrogels, such as poor retention, brittleness, and dehydration, making CTFG@S particularly suitable for use on joints and other anatomical regions with high mobility (Figure 12A) [138].

Complementing this list, Li et al. formulated a chitosan/cellulose nanofiber/tannic acid (CS/CNF/TA) hydrogel, demonstrating potent antibacterial activity and superior tissue healing results. The hydrogel promoted complete wound closure and hair regrowth within 14 days, outperforming the commercial Tegaderm dressing. Histological evaluation revealed accelerated re-epithelialization, reduced epidermal thickness, enhanced collagen deposition, and increased CD31 expression, indicating robust neovascularization and dermal remodeling (Figure 12B). This construct is therefore highly effective for the treatment of infected wounds, combining anti-infective action and regenerative support [139].

To replicate the layered architecture of native skin, a bilayer electrospun dressing was developed from a base layer of bioactive nanofibers (Pec/SPI/P) and a protective top layer of cellulose microfibers. This structure allows for sustained release of phenolic compounds, enhanced keratinocyte adhesion and migration, and significantly increased angiogenic activity, as demonstrated by CAM tests and in vivo rat models. The Cel/Pec-SPI-P dressing enabled complete epidermal regeneration and keratin layer formation, in contrast to the incomplete healing observed in other groups. The combination of bioactivity and layered mechanical protection makes this system a promising candidate for chronic wound applications, where prolonged treatment and structural support are required (Figure 12(Ci,Cii)) [140].

Continuing their efforts toward controlled healing environments, Yawei et al. proposed a checkerboard-patterned hydrogel system incorporating carboxymethylcellulose (CMC), gelatin, polydopamine (PDA), and silver nanoparticles, with paraffin-based hydrophobic zones. This dual-function configuration exploits Schiff base crosslinking for localized antimicrobial and antioxidant effects, while the paraffin-treated zones facilitate moisture control and fluid direction. This structure allows the dressing to adapt to heterogeneous wound beds, where different regions may require varying levels of absorption, protection, or therapeutic delivery, which is particularly useful in diabetic ulcers or burn sites with irregular exudate distribution [130].

Advanced cellulose composite dressings have also incorporated photothermal therapy for wound healing. Cao et al. developed an injectable photothermal antibacterial BC cryogel dressing modified with aldehyde bacterial cellulose and PDA. When exposed to near-infrared light, it demonstrated rapid hemostatic action and accelerated healing of deep skin wounds [131]. Kun et al. explored a similar concept with a dual-photosensitive cellulose nanofiber-based hydrogel dressing, integrating Prussian blue nanoparticles and responsive polymers. This dressing demonstrated rapid hemostasis, high antibacterial efficacy against drug-resistant bacteria, and was suitable for surgical contexts, particularly in managing infected wounds and preventing secondary injury during dressing removal [132]. Unlike standard surgical dressings, this advanced cellulose-based dressing offers improved functionality and better patient outcomes. The biocompatibility of cellulose dressings has been thoroughly evaluated in in vitro cell culture and in vivo animal model studies [130]. Wei et al. studied BC/soybean protein isolate composites (BC/7S and BC/11S) that consistently release proteins, promoting fibroblast proliferation, collagen expression, and reduced inflammation. BC/11S in particular demonstrated faster wound healing due to higher protein release, highlighting its anti-inflammatory and regenerative benefits. This can be compared to other dressings that may not have such a sustained protein release mechanism, highlighting the importance of this feature in promoting wound healing [75].

**Figure 13:**
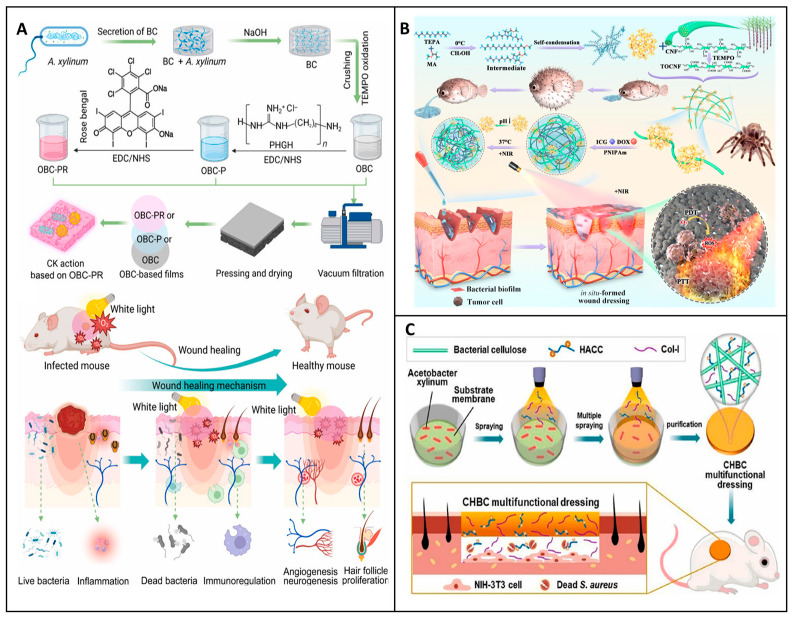
Diagrams illustrating smart cellulose-based dressings for wound healing applications. (**A**) Nature-inspired BC composite film dressing and its mechanism to trap and eliminate bacteria. Reprinted from Ref. [141]. (**B**) In vivo evaluation of multiresponsive, in-situ-forming cellulose nanofibril-based dressing treating complex postoperative infected wound. Adapted with permission from Ref. [142]. Copyright 2021 merican Chemical Society. (**C**) Preparation process of 3D composite scaffolds, BC membrane liquid interface culture, and the process of treating infected wounds in vivo. Reprinted from Ref. [143].

Cellulose-based smart dressings can maintain optimal pH, promoting fibroblast activity and bacterial inhibition [75]. Cellulose-based smart dressings are another area of innovation. Inspired by the “drosera peltate” in nature, Li et al. presented a biomimetic multifunctional cellulose wound dressing film made with oxidized bacterial cellulose (OBC) crosslinked with polyhexamethylene guanidine hydrochloride (PHGH) and rose bengal (RB). The OBR-PC dressing combined photodynamic and chemotherapeutic properties, capturing over 95% of bacteria in minutes. It eliminates pathogens through photodynamic therapy and chemical action while promoting wound regeneration through M2 macrophage polarization and angiogenesis (Figure 13A). This dual-function film offers a rapid and reusable strategy for the management of infected wounds with its textured surface and enhanced blood vessel and nerve regeneration in vivo [141]. Compared with traditional wound dressings that lack such a targeted bacteria capture mechanism, this smart wound dressing offers a more sophisticated approach to wound treatment.

Zhao et al. developed a multifunctional smart CNF-based, in-situ-forming liquid dressing made with cellulose nanofibrils (CNF) grafted with hyperbranched polyamines, forming a platform sensitive to pH, temperature, and near infrared (NIR). This dressing integrates several therapeutic strategies, such as chemotherapy, photothermal therapy, and photodynamic therapy, to treat infected postoperative wounds (Figure 13B). They reported that the dressing efficiently loads and releases doxorubicin and indocyanine green (ICG) and exhibits strong antibacterial activity, tumor inhibition, and biofilm elimination. Additionally, the dressing offered excellent adhesion and tumor-targeting properties, making it ideal for clinical applications, especially on irregular wound surfaces. This is in contrast to wound dressings that may not be as adaptable to different wound geometries and conditions [142].

To further enhance healing speed, Zhou et al. studied a multifunctional BC-based dressing for the rapid healing of infected wounds. The study incorporated Collagen I (Col-I), hydroxypropyltrimethylammonium chloride chitosan (HACC) in a bacterial cellulose (BC) network structure via a novel membrane-liquid interface (MLI) culture and Col-I/HACC/BC (CHBC) culture, as illustrated in Figure 13C. They reported that the dressing exhibited excellent antibacterial properties and cell cytocompatibility in promoting the proliferation and spread of fibroblasts and endothelial cells in just 8 days, even under infected conditions. Improvements in tissue regeneration and collagen expression were also confirmed [143].

Cellulose-based dressings have been shown to be multifunctional and offer significant hemostatic and wound healing benefits. Their superior biocompatibility, cytocompatibility, and hemostatic efficacy have been demonstrated in recent studies. However, despite their promising properties, further clinical trials are essential to validate their safety and efficacy on various wound types. This is necessary to ensure their widespread and appropriate use in healthcare settings. Future research could focus on comparing the performance of different cellulose-based dressings in specific clinical scenarios, such as chronic versus acute wounds, or different anatomical locations. Additionally, studies could explore the long-term effects of these dressings on tissue remodeling and scar formation, which would provide more comprehensive information for their clinical application.

## 6. Clinical Efficacy of Commercial Cellulose-Based Dressings

Commercial cellulose-based dressings have revolutionized hemostasis and wound management, offering effective solutions across diverse clinical settings [144]. These dressings, leveraging the innate properties of cellulose, have been instrumental in controlling bleeding, promoting clot formation, and facilitating wound healing, as detailed in Table 3. In this section, we explore the clinical applications of commercial cellulose-based dressings, referencing relevant studies.

### 6.1. Cellulose-Based Dressings for Hemostasis Available in the Market

Commercial cellulose-based dressings, such as Surgicel^®^, Gelita-Cel^®^, Traumastem^®^, and Interceed^®^, have been extensively employed in trauma and surgical scenarios to achieve hemostasis [145,146,147,148]. These dressings predominantly consist of oxidized cellulose (OC) and oxidized regenerated cellulose (ORC), effectively promoting clot formation and sealing bleeding sites [149].

When it comes to hemostatic efficacy, notable studies have provided valuable insights through comparative analyses. For example, certain cellulose dressings, like Hemopatch^®^, have demonstrated remarkable superiority in controlling bleeding from liver lesions. Others like Surgicel^®^ Original have also been studied in similar contexts [150,151]. However, concerns regarding the risk of foreign body reactions associated with oxidized resorbable cellulose (Gelita-Cel) underscore the importance of careful selection of hemostatic agents [152]. A particularly illustrative study by Uranues et al., 2022, evaluated the effectiveness of two hemostats, namely Hemopatch^®^ and Surgicel^®^ Original, in managing bleeding from liver lesions. The primary endpoint was defined as achieving hemostatic success, which was gauged by reaching grade 0 bleeding on the validated intraoperative bleeding (VIBe) scale after 10 min. The results were quite revealing; Hemopatch^®^ exhibited a significantly higher hemostatic success rate compared to Surgicel^®^ Original. Moreover, Hemopatch^®^ managed to reduce time to hemostasis by 15.9%. Specifically, it outperformed in controlling grade 2 bleeds [153]. Such findings clearly highlight the critical importance of careful selection of appropriate hemostats. This not only optimizes bleeding control during surgical procedures but also contributes substantially to improving patient safety.

Epistaxis, being a frequently encountered medical issue, demands prompt hemostasis to avert potential complications. In this regard, various cellulose-based dressings have emerged as viable options; for instance, Bloodstop^®^ and Traumastem powder. A study was conducted to evaluate the efficacy of topical tranexamic acid and Traumastem powder in comparison to conventional nasal packing methods. The outcomes were rather promising. Traumastem powder, with its notable characteristics of rapid hemostatic action and antibacterial properties, presents a novel approach for managing epistaxis. However, further research is warranted to comprehensively establish its full range of therapeutic benefits [145].

On a different note, Interceed (TC7), which is fabric composed of oxidized regenerated cellulose, has shown considerable promise in reducing postsurgical adhesions. A clinical trial was conducted to evaluate its effectiveness in infertile patients undergoing pelvic sidewall adhesion ablation. The results were significant, as significantly fewer adhesions were observed on the treated sides compared to the control sides. This indicates that the use of TC7 has far-reaching implications for a variety of surgical procedures, including those dealing with severe endometriosis. It thus showcases its potential to improve both patient outcomes and the overall success of surgical interventions [154].

Commercial cellulose-based dressings have firmly established themselves as indispensable assets in the domains of hemostasis and wound management. They offer clinicians a diverse array of solutions for tasks ranging from controlling bleeding to promoting wound healing and preventing postsurgical adhesions. Although the advancements in cellulose-based dressings have already made a substantial positive impact on patient care, continuous research efforts are of utmost importance. This ongoing research should focus on optimizing their efficacy and safety profiles in a wide variety of clinical scenarios. For example, in terms of hemostatic agents, future studies could compare different cellulose-based dressings in more complex surgical scenarios involving multiple bleeding sites or different tissue types. In the context of wound healing, research investigations could explore how variations in the composition or structure of these dressings influence the rate and quality of tissue regeneration. Regarding the prevention of postsurgical adhesions, research could aim to identify optimal application methods and patient populations for whom specific cellulose-based dressings are most effective. Through such comprehensive and comparative research endeavors, the full potential of these dressings can be harnessed to the greatest extent possible to improve patient care.

### 6.2. Cellulose-Based Wound Dressings Available on the Market

In recent years, the employment of cellulose-based dressing materials has seen remarkable advances, with a wide array of commercial products now available on the market [29]. Among these, carboxymethyl cellulose (CMC) wound dressings have gained prominence. Their primary application lies in covering wounds to foster the healing process, although they do not involve the seeding skin cells [109]. However, contemporary literature has emphasized the importance of cell migration properties in enhancing wound healing. This has led to the exploration of integrating fibrin proteins into these dressing [155]. Bacakova and colleagues demonstrated that the incorporation of fibrin resulted in substantial improvements in the cell adhesion and proliferation characteristics of commercially available CMC-based wound dressings, such as Hcel^®^ NaT. Notably, the porous forms of Hcel^®^ NaT exhibited more favorable cell adhesion and proliferation in comparison to their homogeneous counterparts, thus underlining the significance of the material structure [156].

Moreover, commercial wound dressing materials, including Aquacel^®^, have been utilized for controlled delivery of drugs to hasten the wound healing processes. Maver et al. employed Aquacel^®^ for delivery of non-steroidal anti-inflammatory pain-killing drugs, showcasing its biocompatibility with human skin cells and its capacity to maintain water holding and air permeability characteristics. It is essential to note that the efficacy of drug-releasing wound dressings is highly dependent on environmental conditions such as pH and temperature. This emphasizes the necessity for customized formulations to achieve optimal outcomes [157].

Similarly, bacterial cellulose (BC)-based wound dressings, exemplified by products like Biofill and XCell, have emerged as efficacious alternatives for the treatment of burns and chronic ulcers [29]. A study has disclosed the superior performance of Biofill in accelerating the healing process and providing pain relief when contrasted with conventional wound dressing materials [158]. XCell, on the other hand, has demonstrated its effectiveness in managing chronic venous ulcers, further substantiating the versatility of BC-based dressings in addressing diverse wound types [159]. In parallel, commercial cellulose-based dressings like Aquacel^®^ have been extensively evaluated for management of chronic wounds, yielding favorable results in terms of wound re-epithelialization, pain reduction, and ease of use [160].

Clinical investigations comparing various wound dressing options, such as Mepilex^®^ Border Post-Op and Aquacel^®^ Surgical, have furnished valuable insights into optimizing wound healing and preventing postoperative complications. Notably, Mepilex^®^ Border Post-Op has outperformed Aquacel^®^ Surgical in terms of clinician satisfaction, pain reduction, and patient comfort, illustrating the importance of judiciously selecting the most suitable dressing option for optimal results [161]. Additional commercial products containing cellulose include AquaRite Extra CMC™, Biatain^®^, Epiprotect^®^, Fiber 3M™, FibDex^®^, Granugel^®^, Intrasite™, Kerracel™, MedVance^®^, SOLOSITE^®^, Versiva™, Suprasorb X^®^, and XTRASORB^®^. In addition, cellulose often presents as one of the main raw materials in wound pads, further emphasizing its foundational role in modern wound care technologies.

The commercial application of cellulose-based products, especially bacterial cellulose (BC), has transcended the realm of cosmetic skincare to encompass significant strides in hemostasis and wound care. Companies have dedicated resources to research and explore the inherent properties of BC and other cellulose derivatives to develop advanced wound dressings and hemostatic agents. These products are meticulously designed to address complex clinical needs, such as bleeding management, antimicrobial protection, and the promotion of rapid wound healing, making them indispensable in trauma, surgery, and outpatient settings [162].

Several cellulose-based formulations, including oxidized cellulose (OC) and oxidized regenerated cellulose (ORC), have become prevalent hemostatic agents due to their high absorbency, biocompatibility, and ability to promote coagulation. Commercial products such as Surgicel^®^ and Traumastem^®^ are widely used in surgery to control bleeding and improve patient outcomes. Cellulose-based dressings such as Hcel^®^ NaT, Biofill^®^, and XCell^®^ have also shown excellent wound healing properties, particularly for burns, ulcers, and chronic wounds, providing a sterile, non-toxic environment that promotes tissue regeneration [149].

Despite their current widespread application, the growth potential of cellulose-based products remains substantial, as new formulations are continuously being developed to enhance wound care and hemostasis. Further research efforts could be directed toward the integration of antimicrobial agents, the optimization of drug delivery systems, and improvement in the biodegradability and biocompatibility of materials. For instance, in the area of antimicrobial integration, researchers could explore novel compounds or nanoparticles with potent antibacterial activity that can be incorporated into the cellulose matrix without compromising its other beneficial properties. In terms of drug delivery optimization, advanced techniques such as targeted release mechanisms or sustained-release formulations could be investigated to ensure effective and prolonged delivery of therapeutic agents to the wound site. Regarding the improvement of biodegradability and biocompatibility, the use of natural additives or the modification of the cellulose structure at the molecular level could be explored to improve its degradation kinetics and reduce possible adverse reactions in the body. This would not only expand the clinical applications of cellulose-based wound dressings but also contribute to the overall improvement of patient care and treatment outcomes.

**Table 3:** Properties of cellulose-based commercial hemostatic and wound dressings.

Formulation	Properties	Examples	Ref.
Oxidized Cellulose	High water absorption Clot promotion Antimicrobial	Surgicel^®^	[149]
Oxidized Regenerated Cellulose	High mechanical strength Low hemolysis Rapid clot formation	Traumastem^®^, Interceed^®^	[153]
Bacterial Cellulose	Moisture retention Biocompatible	Biofill^®^ XCell^®^	[163]
Bacterial Cellulose with Active Ingredients	Moisturizing Sterile environment Skin hydration	Moisturizer BC mask	[164]
Regenerated Cotton Cellulose	Rapid clot initiation Optimal moist environment Gel-like subtract	Bloodstop^®^	[165]
Carboxymethyl Cellulose (CMC) based	High absorbency Manage high moderate Chronic wound	Fiber 3M™, MedVance^®^ AquaRite Extra CMC™ Hcel^®^ NaT	[25,156]
Sodium Carboxymethyl Cellulose Blend	Antimicrobial management of exudate High absorbency Promotes angiogenesis and autolytic debridement	Suprasorb X^®^, Granugel^®^ Aquacel^®^	[157,166]

### 6.3. Clinical Trials and Therapeutic Evidence

An expanding body of preclinical and clinical research has validated their efficacy in the treatment of chronic wounds, diabetic ulcers, burns, and donor site injuries. Products such as Biofill^®^, XCell^®^, Dermafill™, AquaRite Extra CMC™, Suprasorb X^®^, and FibDex^®^ have demonstrated strong wound healing potential. For instance, Koivuniemi et al. reported that nanofibrillar cellulose hydrogels, while matching synthetic polylactide-based dressings in healing time and pain levels, led to better long-term scar quality and long-term skin elasticity [167].

Similarly, a randomized controlled trial conducted by Alvarez et al. demonstrated that BC-based dressings (Suprasorb X^®^) significantly outperformed conventional non-adherent dressings in the treatment of venous leg ulcers. The cellulose-treated group showed faster autolytic debridement, earlier granulation (25 versus 36 days), faster re-epithelialization (36 versus 50 days), and complete pain relief by week 7. Although overall healing time was not significantly different, the study confirmed better wound bed preparation and improved patient comfort with bacterial cellulose [168].

Another recent clinical study highlights the therapeutic potential of nanocellulose-based dressings in pediatrics (Annika Resch et al.). Nanocellulose-based dressings were applied to children (aged 0–16 years) with second-degree burns covering 1–10% of the total body surface area. Results showed complete re-epithelialization occurred within 7–17 days, with over 95% of wounds healed in 13 patients by day 10 [7].

The ability to leave the dressing in place for up to 7 days minimized hospital visits and stress, which is particularly crucial in pediatrics. The moist environment created by the nanocellulose-based dressing promoted optimal healing, with clinical outcomes comparable to standard care, but offering improved treatment compliance, greater comfort, and potential cost reduction.

Another promising example is AquaRite Extra CMC™, a carboxymethylcellulose-based dressing with strong gelling and absorbent properties. Its ability to manage exudate, trap bacteria, and facilitate painless removal makes it suitable for a wide range of wounds, including pressure ulcers and surgical injuries [25,156,169].

Beyond their use in chronic wounds and burns, cellulose-based materials have demonstrated significant effectiveness as hemostatic agents in clinical and surgical settings. Oxidized cellulose (OC), a chemically modified form of native cellulose, and oxidized regenerated cellulose (ORC) have long been utilized in hemostatic dressings due to their high absorbency, strong mechanical integrity, and bioactive surface chemistry. These materials rapidly absorb blood, forming a gelatinous matrix that promotes clot formation, facilitates vasoconstriction through localized acidification, and activates intrinsic and extrinsic coagulation pathways. Their antimicrobial properties also help prevent infection at the wound site [170].

Among the most well-established recognized ORC products is Surgicel^®^, widely used in surgical procedures [149]. However, clinical evidence, such as the study by Amit et al., has cautioned that while Surgicel^®^ is effective for intraoperative hemostasis, its routine use in certain surgeries like thyroidectomy may pose risks of inflammation or delayed healing [171]. To improve upon traditional ORC materials, novel composites have been developed. For example, ORC blended with chitin/chitosan has demonstrated enhanced antimicrobial and hemostatic effects in chronic animal infections [172], while an ORC-modified chitosan sponge showed rapid and effective bleeding control in mouse tail vein and rabbit femoral artery models [173]. Similarly to ORC blended with collagen/silver, ORC showed good management of skin graft donor site wounds, particularly in patients with known risk factors for wound healing [174].

A comparative study by Lewis et al. highlighted the superior performance of oxidized non-regenerated cellulose (ONRC) over ORC. Using in vivo porcine and rabbit models, the study demonstrated that ONRC-based Traumastem^®^ (Biostar) achieved faster hemostasis and comparable antimicrobial efficacy to Surgicel^®^. These results suggest that ONRC, due to its unregenerated fibrous structure, may offer improved surface interaction for platelet adhesion and clot stabilization [175]. Overall, cellulose-based hemostatic dressings, particularly oxidized forms, continue to evolve through compositional and structure modifications. Collectively, these clinical findings confirm that cellulose-based dressings not only match but often exceed conventional synthetic dressings in terms of rapid bleeding regulation, moisture control, infection prevention, and wound healing efficacy.

### 6.4. Comparison with Synthetic, Hydrocolloid, Hydrogel, Foam, Alginate, and Protein-Based Dressings

Synthetic polymer dressings, such as those composed of polyurethane (PU), polycaprolactone (PCL), polyethylene glycol (PEG), and poly(lactide-co-glycolide) (PLGA), dominate the wound care market [176]. Their customizable degradation profiles, high mechanical strength, and drug delivery capabilities make them appealing for advanced wound therapies (Table 4). Products like PU foams and PEG-based hydrogels are widely used and backed by extensive clinical trial data, especially in treating diabetic foot ulcers and surgical wounds [177].

Beyond moisture management, synthetic polymers also play a vital role in hemostasis. Polymers like α-cyanoacrylates, PEG, and polyesters have been designed for hemostatic sealants due to their ability to form strong adhesive barriers upon contact with blood. Cyanoacrylates were among the earliest materials used as wound sealants, with FDA-approved products such as Dermabond^®^, Omnex^®^, Glubran^®^, and IFABond^®^ emerging as clinical staples [178]. These agents polymerize rapidly through anionic reactions upon exposure to moisture, forming covalent bonds with skin tissue. However, their use is limited by post-polymerization brittleness and the release of cytotoxic byproducts like formaldehyde and cyanoacetic acid, which can induce inflammation [178]. These synthetic materials are widely validated in randomized controlled trials (RCTs) and carry regulatory familiarity (e.g., FDA Class II/III approval), which underpins their dominance in the global wound care market. However, sustainability concerns, limited biodegradability, and higher production costs compared to natural biopolymers are pushing the field toward greener alternatives [179].

Hydrogel-based wound dressings are widely adopted for their high water content (80–90%), which allows them to maintain a moist wound environment, promote autolytic debridement, and reduce wound bed temperature by up to 5 °C. They also permit gas exchange and minimize patient discomfort during dressing changes. However, hydrogels offer limited antibacterial protection and often require frequent replacements (Table 4). Thus, they are typically combined with antimicrobial agents when managing infected wounds [180,181].

Foam dressings are favored for treating wounds with heavy exudation. They offer excellent absorption, thermal insulation, and breathability, which helps regulate wound moisture and promote healing. Clinical studies have demonstrated the superior fluid-handling capacity of certain foam brands (e.g., Biatain^®^ vs. Allevyn^®^), which can reduce dressing change frequency and treatment costs. Although foam dressings may include antimicrobial agents (e.g., povidone-iodine), their primary benefit lies in exudate management rather than antibacterial activity [182,183].

Hydrocolloid dressings are moisture-retentive, semi-occlusive systems, composed primarily of pectin or gelatin, and are valued for their absorptive and moist wound-healing environment, but they often suffer from drawbacks like malodor, residue on removal, and limited breathability (Table 4). Upon contact with wound exudate, they form a gel that promotes autolytic debridement and maintains a moist healing environment. These dressings provide a protective barrier against bacteria without requiring secondary dressings [184]. They are ideal for non-infected, low-to-moderate exudate wounds but are unsuitable for infected or heavily draining wounds due to their occlusive nature. While effective in accelerating healing, their strong adhesion may cause trauma upon removal. Clinical studies confirm their efficacy, particularly in enhancing inflammation modulation and bacterial inhibition through localized pH control [185].

Meanwhile, protein-based dressings, such as those derived from collagen, fibrin, keratin, and silk fibroin, closely mimic the extracellular matrix, promoting angiogenesis and tissue regeneration (Table 4). However, their usage is restricted by high production costs, potential immunogenicity, and ethical considerations tied to animal-derived sources [186,187,188]. Among biopolymers, alginate-based dressings have gained significant clinical and commercial traction thanks to their biocompatibility, hydrophilicity, and excellent fluid absorption. Upon contact with wound exudate, sodium ions in alginate are exchanged with calcium ions, forming a gel that maintains a moist wound environment and facilitates autolytic debridement (Table 4), making them dressings that are well-suited for moderate to heavily exuding wounds but unsuitable for dry wounds, third-degree burns, or wounds with exposed bone due to their risk of adherence to healing tissues and potential inhibition of keratinocyte migration [170].

Nevertheless, some studies suggest that specific alginate formulations promote macrophage activation and accelerate healing. Alginate-based dressings are available in a wide variety of forms, including hydrogels, foams, wafers, nanofibers, and membranes, and have been successfully integrated with various drugs to enhance therapeutic outcomes. Commercial examples include Kaltostat^®^, Melgisorb^®^ Plus, Biatain^®^ Alginate, and 3M™ Tegaderm™ Alginate Dressing, the latter of which blends calcium alginate with carboxymethylcellulose (CMC) and guluronic acid to enhance absorbency, support autolysis, and allow painless removal [189].

In contrast, cellulose-based dressings offer a balanced profile, combining the biofunctionality of protein-based dressings with the cost-efficiency and environmental benefits of polysaccharides (Table 4). They support wound healing processes such as fibroblast proliferation, angiogenesis, and re-epithelialization, while offering better sustainability and easier scalability than many synthetic or protein-based systems [190].

Despite their clinical validation, cellulose-based dressings are still classified under moderate regulatory categories (e.g., FDA Class II/III approvals) and have no widespread clinical adoption. However, this landscape is evolving. The rise of commercial cellulose-based products such as AQUACEL^®^, Biatain^®^ Fiber, Kerracel™, XTRASORB^®^, Versiva™, and Epiprotect^®^ reflects a shift in both industry focus and clinical interest. Additionally, cellulose integration into hybrid products with alginate, hydrocolloids, hydrogel systems, and synthetic polymers offers enhanced performance through synergistic effects. As more clinical data accumulate and sustainability becomes a strategic priority in healthcare procurement, cellulose-based dressings are increasingly penetrating mainstream wound care segments [29].

**Table 4:** Summary of the mechanisms, advantages, and limitations of commercial dressings vs. cellulose-based dressings.

Category	Composition	Mechanism of Action	Clinical Application	Benefit	Limitation	Commercial Product	Ref.
Synthetic based	PEG, PVA, PU Polyesters α-cyanoacrylates	Sealant formation Vessel occlusion Crosslinking	Trauma, surgical bleeding controlled drug delivery	Customizable Scalable Fast hemostasis	Brittle gels, inflammation risk, poor biodegradability	Dermabond^®^, Omnex^®^, Glubran^®^, Glubran2^®^, IFABond^®^, PVA-Chitosan pad	[176,177,178]
Hydrocolloid based	Sodium CMC, gelatin, pectin, sodium alginate	Gel formation, moisture retention, autolysis stimulation	Low–moderate, exudating wounds, ulcers, pressure injuries	Moist environment, self-adherent, bacterial barrier	Not for infected wounds, trauma during removal	DuoDERM^®^, Comfeel^®^, Tegasorb^®^, Granuflex^®^, Hydrocoll^®^	[184,185]
Hydrogel based	PEG-based hydrogels natural/synthetic blends	Hydration, cooling, autolytic debridement	Burns, dry wounds, necrotic wounds	High moisture, soothing, drug- loaded capacity	Poor absorbency, not ideal for heavy exudate	Clearsite^®^, Intrasite^®^, DermaSyn^®^, NuGel^®^, AquaClear^®^, SOLOSITE^®^	[180,191,192]
Foam based	Polyurethane Silicone-coated	Exudate absorption Mechanical protection	Moderate–heavy, post-op wounds, pressure injuries	High absorbency, cushioning, non- adherent top layers	Risk of maceration, May require secondary dressing	Mepilex^®^, Allevyn^®^, Lyofoam^®^, PolyMem^®^, Biatain^®^	[182,183]
Protein based	Collagen, Fibrin, Keratin, Silk fibrin/sericin	Cell migration Growth factor delivery Enzymatic degradation	Chronic wound Burns wound Diabetes ulcers	Biocompatible, Promotes granulation,re-epithelialization	Costly, Sometimes antigenic	Promogran^®^, EpiFix^®^, FIBRACOL^®^, OASIS^®^ Biostep^®^, MatriStem^®^,	[186,187,188]
Alginate based	Calcium/sodium Alginate from brown seaweed	Ion exchange (Ca^2+^ for Na⁺) Gel forming matrix	Moderate– heavy wounds, surgical wounds, bleeding ulcers	High absorbency, Promotes clotting	Not for dry wounds, can leave residue	Kaltostat^®^, Algisite^®^ Tegaderm Alginate^®^, Sorbsan^®^, Melgisorb^®^	[170,189]
Cellulose based	Bacterial cellulose	Moisture retention biocompatibility	Diverse burns, chronic wounds, ulcers,	Abundant, non-toxic, customizable, porosity,	Slower bio resorption	Biofill^®^, XCell^®^,	[168]
	Oxidized cellulose (OC)	Hemostasis	bleeding wounds Surgical intervention	sterile, non-toxic, biodegradable	Some need Secondary dressings	Surgicel^®^, Traumastem^®^	[7]
	Oxidized regenerated cellulose (ORC)	Hemostasis, promotes granulation	bleeding wounds Surgical bleeding	biodegradable, drug delivery	Some allergy reaction	Surgicel^®^, Traumastem^®^	[149,171]
	CMC derivatives	Physical protection	Trauma wound, surgical sites, outpatient and trauma care	diverse product types	Variable clinical performance depending on formulate	Aquacel^®^,	
						AquaRite	[193]
						Extra CMC™	
						Hcel^®^ NaT	[25]

### 6.5. Cost-Effectiveness and Sustainability Analysis

One of the strongest arguments in favor of cellulose-based wound dressings lies in their economic and environmental sustainability. Derived from renewable sources like plant biomass, agricultural waste, or microbial fermentation, cellulose is both abundant and inexpensive. Its extraction and processing require less energy and fewer chemical reagents than the synthesis of materials like PU, PEG, or PCL. Clinically, cellulose dressings can reduce healthcare costs by accelerating wound healing, decreasing dressing change frequency, and lowering infection rates, which in turn reduce hospitalization duration and associated medical expenses. Furthermore, life cycle assessments (LCAs) reveal that cellulose-based products have a significantly lower environmental footprint, generating less non-biodegradable waste and greenhouse gas emissions compared to petroleum-derived synthetics. As global healthcare systems pivot toward environmentally responsible solutions and circular economy models, cellulose dressings offer a future-ready alternative that aligns with both clinical efficacy and green innovation [194].

## 7. Emerging Trends and Future Prospects

Cellulose-based materials have already established themselves as highly promising and represent an innovative frontier in wound care and hemostatic applications. Their inherent biocompatibility, biodegradability, and eco-friendly nature have laid a solid foundation for sustainable alternatives to traditional synthetic counterparts. However, the road ahead is laden with opportunities for even more revolutionary advances. One of the most promising areas lies in the enhancement of mechanical performance. By leveraging innovative techniques such as precise fiber orientation control and the incorporation of advanced reinforcing agents, including nanoparticles with unique properties, inorganic biomaterials with enhanced functionality, and biopolymers with tailored characteristics, we can engineer cellulose-based composites that not only exhibit superior durability and handling capabilities but also integrate antimicrobial properties. This multifaceted approach will redefine the standards for advanced materials for wound care, enabling them to withstand the rigors of diverse clinical scenarios [195]. The exploration of porosity optimization in cellulose scaffolds represents another frontier of innovation. By increasing porosity, we can create a highly conducive environment for cell seeding and tissue integration. This will not only accelerate the wound healing process but also open up new possibilities in tissue engineering, where the interaction between cells and the scaffold is of utmost importance.

To address the challenge of slow biodegradation, a slew of innovative strategies is on the horizon. Chemical modifications, such as the incorporation of functional groups or copolymers, can be fine-tuned to achieve a delicate balance between accelerated degradation under physiological conditions and preservation of mechanical integrity. The integration of cellulolytic enzymes into the material matrix is a game-changing concept that allows for targeted degradation after application, ensuring that the material’s lifespan aligns with the wound healing timeline. Additionally, the development of hybrid systems combining cellulose with faster-degrading polymers, such as poly(lactic-co-glycolic) acid (PLGA), offers a novel solution to achieve a precisely controlled degradation rate, thereby optimizing the material’s performance throughout the wound healing process [196]. In the realm of drug delivery systems, the future holds the promise of unprecedented innovation. The development of controlled release mechanisms for bioactive molecules will enable us to deliver therapeutic agents more precisely and sustainably, accelerating wound healing and effectively managing infections. Emerging nanotechnologies, such as the incorporation of silver or other antimicrobial nanoparticles and the utilization of electrospun nanofibers, are poised to overcome the limitations of mechanical strength and infection control. These nanoparticles can be designed to release their antimicrobial payload in a controlled fashion, providing long-term protection against infections while maintaining the integrity of the dressing.

The activation of the intrinsic coagulation pathway by cellulose dressings is an area ripe for exploration. Future research should focus on unraveling the role of modified cellulose in platelet aggregation and coagulation factor activation, exploiting this knowledge to develop wound dressings with improved hemostatic efficacy. This could involve designing cellulose derivatives with specific chemical moieties that interact with the coagulation cascade in a more targeted manner [197]. Green chemistry approaches for the production of cellulose wound dressings are not only environmentally friendly but also offer a sustainable solution to reduce medical waste. By aligning with the global trend toward eco-friendly healthcare materials, we can ensure that the production process is as beneficial to the planet as the dressings are for patients. The application of cellulose-based dressings, particularly bacterial cellulose (BC), has shown great promise in various wound types. However, to bridge the gap between preclinical studies and clinical practice, long-term assessments of in vivo biocompatibility, toxicity, and biodegradability are essential. This will involve the use of advanced animal models with extended monitoring periods and the development of non-invasive techniques to evaluate material performance in real time [25]. Furthermore, certain cellulose composites, such as oxidized cellulose, may lead to delayed absorption or healing complications. Allergic reactions to components like chitosan also require attention [198].

To mitigate the challenges related to delayed absorption and wound healing complications, innovative strategies are being devised. Modifying the structure of cellulose through partial oxidation or enzymatic treatments can optimize degradation under physiological conditions, ensuring that the dressing is absorbed in a timely manner without compromising the wound healing process. Blending cellulose with faster-degrading polymers, such as polylactic acid (PLA) or polycaprolactone (PCL), provides an additional level of control over the degradation rate. Incorporating drug-loaded composites with anti-inflammatory agents or bioactive molecules can address complications resulting from delayed absorption, promoting a more favorable wound healing environment [199]. In the pursuit of improved mechanical resilience and long-term antimicrobial effectiveness, the development of sustained-release systems embedded within cellulose matrices is a key area of focus. These systems can provide a continuous and controlled release of antimicrobial agents, such as nanoparticles or antibiotics, ensuring long-term protection against infections. Bioactive coatings with antimicrobial properties, such as quaternary ammonium compounds or essential oils, can be applied to the surface of the dressing, further enhancing its antibacterial capabilities.

The incorporation of photosensitive materials for photodynamic therapy (PDT) represents a cutting-edge innovation. By integrating materials that can be activated by specific wavelengths of light, we can achieve on-demand antimicrobial effects, providing a powerful tool to combat infections. This approach not only offers a more targeted and effective treatment but also reduces the risk of antibiotic resistance. To make cellulose-based dressings more accessible and cost-effective, it is essential to reduce production costs through green chemistry and automated manufacturing. The exploration of solvent-free processes and eco-friendly extraction methods will not only support large-scale sustainable applications but will also contribute to a more environmentally conscious healthcare industry. The future also holds the promise of smart dressings that can adapt to the dynamic needs of the wound. These dressings, which respond to specific wound conditions such as pH or temperature, will release therapeutic agents precisely when required, optimizing the healing process. Personalized dressings, created using advanced fabrication techniques like 3D printing or electrospinning, will revolutionize patient care by tailoring the dressing to the individual’s unique wound characteristics.

Biohybrid dressings, which combine living or stem cells with cellulose matrices, are set to transform the treatment of chronic wounds. By harnessing the regenerative potential of cells, these dressings can promote tissue regeneration in a way that was previously unimaginable. However, to realize the full potential of these innovative dressings, comprehensive clinical trials are essential to establish their safety, efficacy, and regulatory compliance. From an industrial standpoint, research should concentrate on scalable and economically viable production methods to make cellulose-based dressings competitive with traditional wound care materials. Enhanced surface modification techniques could resolve issues related to hydrophilicity, further expanding their application scope. In conclusion, the future of cellulose-based materials in wound care is filled with boundless possibilities. By embracing innovation and collaboration across multiple disciplines, we can unlock the true potential of these materials and usher in a new era of wound care that is more effective, sustainable, and patient-centric.

## 8. Conclusions

Cellulose-based materials have heralded a revolutionary breakthrough in the domains of hemostasis and wound healing, proffering sustainable and multifaceted solutions to the contemporary conundrums faced by the healthcare sector. These wound dressings capitalize on the remarkable attributes of cellulose, including its outstanding hemostatic capabilities, excellent biocompatibility, and remarkable adaptability, thereby effectively catering to the crucial requisites of wound care. Notably, functionalized cellulose-based dressings and composite architectures have evinced remarkable effectiveness in staunching bleeding and expediting the wound healing process, thus establishing cellulose-based constructs as pivotal elements in blood-interaction applications.

Nevertheless, the path toward their seamless integration into clinical practice is fraught with challenges. The intricate synthesis and fabrication procedures, exorbitant costs, and relatively narrow scope of applications, predominantly confined to cutaneous wounds, present substantial impediments. Overcoming these limitations necessitates a two-pronged approach: driving forward chemical design and manufacturing technologies to optimize production efficiency and augmenting their functional diversity for a wider array of biomedical applications. Innovations such as the incorporation of inorganic constituents, the refinement of surface modification methodologies, and the infusion of nanotechnology will be indispensable in surmounting these obstacles.

Emerging novel cellulosic composite dressings—for instance, those integrating bioactive nanoparticles, bioengineered proteins, or pro-angiogenic agents—are emerging as the vanguard of next-generation hemostatic materials. Integrated bio-inspired designs, such as emulating the hierarchical architectures of blood vessels or leveraging stimuli-responsive characteristics (e.g., pH- or temperature-triggered expansion), present promising avenues for enhancing both efficacy and functionality. Future research endeavors should also be centered on the clinical translation of these materials, tackling challenges like cost-effective manufacturing, ensuring long-term storage stability, and devising strategies for large-scale production. As the impetus to enhance the performance and availability of cellulose-based wound dressings gains momentum, these materials are set to offer versatile, patient-centered solutions that alleviate healthcare burdens and enhance clinical outcomes. Ultimately, a strategic exploration of cellulose composites and functionalization techniques will unleash the latent potential of these materials, cementing their status as indispensable assets for next-generation wound care and broader biomedical innovations.

## Figures and Tables

**Table 1:** Mechanisms and therapeutic roles in hemostasis and wound healing of cellulose-based dressings.

Type of Wound	Cellulose-Based Dressing	Mechanism of Action	Therapeutic Role	Ref.
Abrasions	Oxidized cellulose	Matrix for fibrin deposition Platelet aggregation	Reduces infection Re-epithelialization	[21,22]
Lacerations	Sponge-based hemostatic	Physical barrier Activation platelets Red blood cell activation	Accelerates clotting Tissue repair	[23,24]
Surgical Incisions	Carboxymethyl cellulose (CMC)	Formation of gel-like structure upon contact with exudate	Maintains moisture Reduces infection Promotes granulation	[25]
Burn Wounds	Nanocellulose-based hydrogels	Mimicking structure of extracellular matrix	Reduces pain and infection Tissue regeneration and recovery	[26,27,28]
Pressure Ulcers Chronic Wounds	Bacterial cellulose	Three-dimensional subtract cell fixation Mimicking the extracellular matrix	High fluid retention Tissue regeneration Angiogenesis Collagen formation	[29,30]
Chronic Wounds	Cellulose acetate	Modulates immune response Maintains a moist environment	Antibacterial Tissue repair and skin cell proliferation	[31,32]

## Data Availability

No new data were created or analyzed in this study. Data sharing is not applicable to this article.
